# Midwife-led pandemic telemedicine services for maternal health and gender-based violence screening in Bangladesh: an implementation research case study

**DOI:** 10.1186/s12978-023-01674-0

**Published:** 2023-08-29

**Authors:** Amirul Islam, Farida Begum, Anna Williams, Rabeya Basri, Rowsan Ara, Rondi Anderson

**Affiliations:** 1 United Nations Population Fund, Dhaka, Bangladesh; 2 Data, Design + Writing, Portland, USA; 3 Bangladesh Directorate General of Nursing and Midwifery, Dhaka, Bangladesh; 4 United Nations Population Fund, Aleppo, Syria

**Keywords:** Telemedicine, Remote healthcare, Midwifery, COVID-19, Antenatal care (ANC), Postnatal care (PNC), Gender based violence (GBV)

## Abstract

**Background:**

The COVID-19 pandemic disrupted maternal and newborn health services in Bangladesh, exacerbating the large gaps in service utilization that existed prior to the pandemic. As part of its response, Bangladesh initiated remote antenatal and postnatal care telemedicine services led by midwives in 36 sub-district hospitals across five of Bangladesh’s 64 districts. Gender-based violence screening and referral were integrated into the service to address a reported rise in violence following the country’s pandemic lockdown.

**Methods:**

Mixed-methods implementation research was used to develop an intrinsic case study describing the design and implementation of the telemedicine program. Qualitative analysis comprised document review, key informant interviews, and focus group discussions. Quantitative analysis employed an interrupted time series analysis with segmented multi-variate regression to compare maternity care service use trends before and after implementation. Poisson regression analysis was used to examine the trend in number of gender-based violence remote screenings, sessions held, and cases identified.

**Results:**

A statistically significant change in trend for onsite antenatal and postpartum care as well as women seeking care at the hospital as a result of postpartum hemorrhage arising in the community was observed following the introduction of telemedicine. Facility births and cases of eclampsia appropriately identified and managed also had significant increases. In addition, over 6917 women were screened for GBV, 223 received counseling and 34 referrals were made, showing a statistically significant increase in frequency over time following the implementation of the telemedicine program. Challenges included that not all midwives adopted GBV screening, some women were reluctant to discuss GBV, there was an unanticipated need to introduce a patient visit scheduling system in all intervention hospitals, and many women were not reachable by phone due to lack of access or network coverage.

**Conclusions:**

Maternal health and gender-based violence telemedicine led by midwives was an effective, low-cost intervention in Bangladesh for addressing pandemic and pre-pandemic gaps in service use. Other low and middle-income countries planning to implement remote maternal health interventions via midwives should consider whether a patient visit scheduling system needs to be introduced, as well as limitations around mobile phone access and connectivity. Future research should include care quality oversight and improvement, and a more well-informed strategy for facilitating effective GBV screening.

**Supplementary Information:**

The online version contains supplementary material available at 10.1186/s12978-023-01674-0.

## Plain language summary

To support the continuation of sexual and reproductive health services following pandemic lockdowns, Bangladesh introduced a midwife-led telemedicine program. Through the program, midwives who were already employed within the health system delivered remote antenatal and postnatal care, including gender-based violence screening and referral. The program operated in 36 sub-district hospitals across five of Bangladesh’s 64 districts. Intrinsic implementation research was used to develop a case study describing the design and implementation of the telemedicine program. Qualitative and quantitative methods comprised document review, key informant interviews, focus group discussions, and service use trends. Analysis of the data identified a statistically significant trend increase for most maternity care services. Although they did increase significantly over time, referrals for GBV were less than expected, which may have been related to some midwives not screening for GBV, and/or that many women were reluctant to discuss GBV. In addition, there was an unanticipated need to introduce a patient visit scheduling system in all intervention hospitals, and many women were not reachable by phone due to lack of access or network coverage. In spite of this, 6197 women were screened for GBV. Of those, 223 received counseling and 34 received referrals. Overall, telemedicine led by midwives was an effective, low-cost intervention for maternal health, and a step toward stronger GBV response in Bangladesh. Other low and middle-income countries planning to implement remote maternal health interventions via midwives should consider what is needed to facilitate comfort for both providers and women as related to GBV screening, as well as practical issues regarding introducing scheduling systems and limitations of mobile phone access and connectivity.

**Supplementary Information:**

The online version contains supplementary material available at 10.1186/s12978-023-01674-0.

## Introduction

The COVID-19 pandemic disrupted maternal and newborn health services worldwide [1]. In Bangladesh, national lockdown orders extended from March 26 to May 30, 2020 [2]. By April 2020, antenatal care (ANC) service use had declined in comparison to the previous year by as much as 50%, and reports of GBV during lockdown were increasing [3–5]. Facility birth, postnatal care (PNC), and family planning (FP) service patterns followed similar trends [6]. While service use began to steadily increase shortly after, by July 2020, ANC utilization was still 20–25% lower than it had been the previous year. By September 2020, ANC, PNC, facility birth and FP service use had still not fully recovered [6].

The decline in service utilization below expected levels compounded pre-existing limitations. National survey data from 2017 and 2018 showed that only 47% of pregnant women attended four ANC visits, and only 18% received quality ANC during their most recent pregnancy [7]. In addition, half of all deliveries took place at home without skilled birth attendants and only slightly over half of district hospitals met basic emergency obstetric care criteria [7, 8].

The World Health Organization (WHO) recommends that all women are screened for intimate partner violence (IPV) during antenatal care [9]. Intimate partner violence during pregnancy is thought to be 35% higher than for non-pregnant women. In addition to increased risk of preterm birth, low birthweight, and small size for gestational age, in some contexts it may be the leading cause of death for pregnant women and their newborns [10, 11]. Bangladesh is known to have high rates of GBV, including IPV, before the COVID-19 pandemic, between 60 and 87% of women reported experiencing IPV, one of the highest rates of IPV globally [12]. Reports of increased GBV during COVID 19 were published both globally and in Bangladesh [12, 13]The increases were thought to be due to a variety of factors with loss of income and close quarters during quarantine being the most significant within the Bangladesh context [14]

This program was initiated by the Director General of Nursing and Midwifery, with the support of UNFPA in response to the COVID 19 pandemic. Prior to this intervention there were no established routine, provider-led, or specifically midwife-led, maternal health or GBV services using telecommunications technology–hereafter referred to as telemedicine–in Bangladesh. Various hotlines responding to calls from pregnant women, and awareness raising texting programs had been supported by the government in prior years. Example are the Aponjon program which targets expecting women with pregnancy health text messages but does not provide phone conversations [15]. Another national hotline program had equipped 28 hospitals and over 4000 local-level information centers with a service that allows the public to reach doctors by mobile phone for health advice as opposed to a standard ANC or PNC visit. However, neither provide ANC and PNC packages of care. The telemedicine program described in this intervention was an addition to these existing programs and the first to provide a standard ANC and PNC package remotely [16].

The national pandemic response was the motivator for this first national midwife-led telemedicine program. It was introduced into five project supported districts in Bangladesh in December 2020. Its aims were to enable continued sexual and reproductive health services for pregnant women–including GBV screening and referral–despite pandemic-related movement restrictions, service limitations and fears of COVID-19 transmission in healthcare settings.

This implementation research paper discusses the process of designing and rolling out the initiative and analyzes one year of service statistics.

## Methods

The study was part of a larger project approved by the Ministry of Health and Family Welfare and funded by the Swedish International Development Cooperation Agency (SIDA) with ID BGD10MWC. The objective of this research was to understand the acceptability, practicality, implementation, and outcomes of midwife-led telemedicine services on antenatal and postnatal care, inclusive of gender-based violence screening. An intrinsic case study format was used employing mixed methods in order to synthesize and communicate project monitoring information [17]. Data collection was led by an anthropologist trained in qualitative research who oversaw project monitoring and reporting. The PhD author who has a qualitative research background supervised data collection and analysis.

Qualitative analysis comprised document review, key informant interviews, and focus group discussions to develop an understanding of the implementation experience. Program documentation consisted of internal and external monitoring reports and qualitative data collection. Interviews and focus groups were intended to deepen understanding of experiences and motivators of the involved participants. Eighteen interviews and two focus groups were held with midwives, managers, and service users by the lead author between October and December of 2021 as part of project monitoring.

Participants were chosen based on their involvement with the program, and their ability to shed light on successes, facilitators, and barriers. The sample for healthcare provider interviews was a random selection of those known to be involved in the intervention in the different locations. Of the participants, ten were national and district-level managers and eight were midwives providing remote services. Service users at rural public health facilities are known to be socio-economically homogeneous. More than 90% belong to the lowest wealth quintiles, and are married, in the 20–29 year age bracket, and Muslim [7]. Therefore, convenience sampling was used with all women at facility sites who had participated in the telemedicine program and agreed to participate during researcher visits. Two focus group discussions and eight additional interviews were held with pregnant and post-partum service recipients.

As part of program monitoring, an interview guide was developed based on the guidelines for ANC and PNC during the COVID-19 pandemic. The guide was reviewed and authorized by researchers serving within government and within a leading national research institution. The guide was designed to gather information about the implementation process, including challenges encountered, solutions, and feelings and experiences with the program. Interviews and focus groups were conducted in Bangla, which was the native language for both the researcher and the participants. Notes were kept in Bangla of each session, the notes were later translated to English by the researcher. Interviews with healthcare providers and managers were held over the phone. An additional focus group and eight interviews with service users were conducted onsite at the sub-district hospitals. Interviews and focus groups were conducted until data was saturated and minimal new data were emerging [7]. The notes from the interviews and focus groups were analyzed iteratively looking for themes and provided context to the program documentation and service statistics. Nine themes were identified (Table 1).

**Table 1:** Themes from the qualitative analysis

Theme	Description
Scheduling system initially challenging	Managers—No existing system for scheduling patient visits. There was a learning curve for accepting its need and using it effectively
Women not reachable	Midwives—Women do not always have phones, many unreachable
Midwives like checklists	Midwives—Midwives stated that checklists were helpful and had adapted them for onsite care
Midwives busy	Midwives—Added to an already busy workload, would like to have less documentation responsibility
	Managers—Daily scheduling needed to ensure compliance
Client provider relationship building	Midwives—Calling clients keeps them connected
	Managers––Helps to realize existing government initiatives
	Women—Women felt comfortable calling the midwife for problems
More service utilization	Midwives/Managers—Women are more likely to come for services after phone calls
	Managers—Because of the phone calls more women are coming for ANC and PNC
	Women—We know the midwife and feel more comfortable visiting the health center
Avoidance of unnecessary hospital visits	Midwives—Phone calls help women to feel safe at home
	Managers—During COVID 19 telemedicine helps women avoid exposure
	Women—Midwives provided reassurance around common complaints
Linking high-risk women to services	Midwives—Telemedicine is very helpful to identify problems and encourage care
	Women—Midwife provides reminders of danger signs and answers our questions. When needed they encourage us to seek care
Community reluctance to speak about GBV but may be more comfortable on the phone than in person	Midwives—Midwives state women are reluctant to speak about GBV during phone interviews, but still they disclose more on the phone than face-to-face
	Managers—Women are reluctant to talk about GBV face-to-face but more are disclosing using telemedicine

Quantitative data on the implementation of the program, and changes in service utilization after the initiation, were collected to understand the practicality and success of implementation, and the impact of telemedicine on care seeking and utilization. Data were collected from all facilities as part of program reporting, and analysis from existing government databases complemented project data. Quantitative analysis employed an interrupted time series analysis with segmented multi-variate regression to examine the impact of the introduction of the telemedicine program on ANC 1–4, facility births, PNC 1–2, PPH, and eclampsia. Poisson regression analysis was used to examine the trend in number of GBV remote screenings, GBV sessions held, and GBV cases identified. The analysis was performed using the R statistical language [18] utilizing the lme4 package [19] for statistical modeling and the ggplot2 package [20] for generating plots. Statistical significance was determined using a significance level (α) of 0.05. Both project reports and data from the government data collection system, DHIS-2, were analyzed. Quantitative data were sourced from the national health information system (DHIS2) [21] and a separate Google Sheet used for tracking telemedicine service data. Telemedicine service statistics reported numbers of client visits for ANC, facility birth, PNC, FP and GBV screening, identification and referral. The program data were compared with government DHIS2 statistics from the hospitals that introduced the telemedicine program both before and after the pandemic.

## Program description

### Background

The telemedicine intervention arose from a partnership between the Bangladesh Directorate General of Nursing and Midwifery (DGNM) and UNFPA. The partnership supports the government in its introduction of an International Confederation of Midwives (ICM) standard midwifery cadre into the Bangladesh health system. Support to this new cadre of globally standard midwives had been ongoing for seven years prior to the pandemic with multi-donor funding, and by early 2020 had educated 6116 diploma midwives and deployed 1149 midwives into 342 sub-district hospitals. Most midwives have low-middle class backgrounds, and all completed higher secondary education prior to joining the three-year midwifery diploma program. Sub-district hospitals are public secondary-level care facilities that serve populations from the lowest wealth quintiles.

### COVID-19 telemedicine guidance

The *COVID-19 Pandemic UNFPA Global Response Plan* provided the initial impetus for the telemedicine program [22]. Under its umbrella, a *Technical Brief for Maternity Services* with addendum guidance documents for ANC and PNC were released in April and May of 2020 [23, 24] (Additional files 1 and 2). The guidance documents were designed to support the continuation of maternal health services following pandemic-associated service disruptions.

**Fig. 1:**
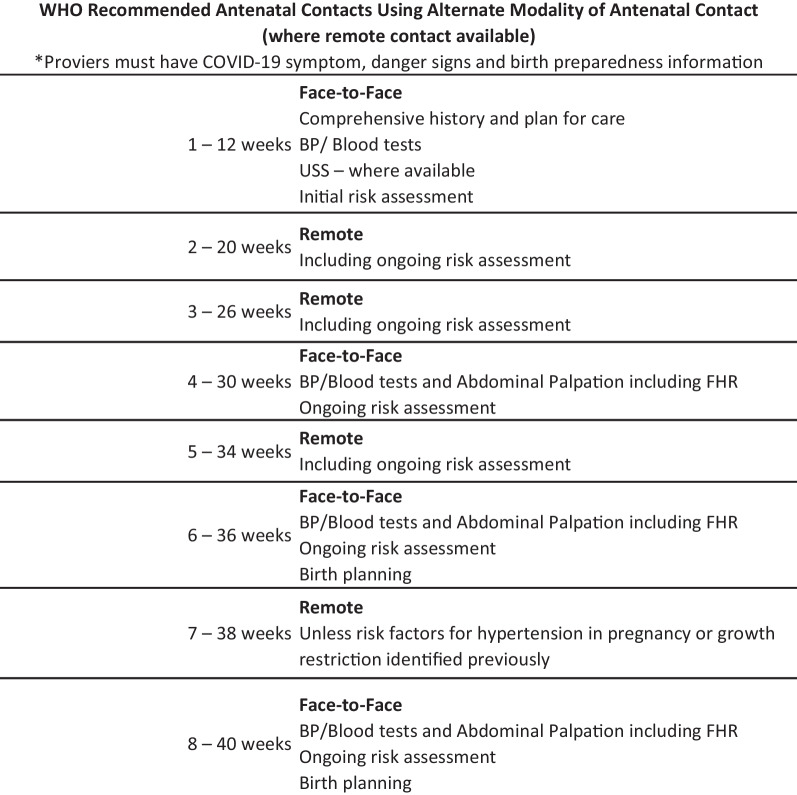
Recommended ANC visit schedule

One key element of the Brief was the inclusion of ANC and PNC using remote contact where possible during the COVID-19 pandemic [23, 24]. Eight ANC visits are recommended according to WHO guidelines with the 1st, 4th, 6th and 8th visits in person and the others via phone or video contact. All midwives followed a standard checklist at every visit to guide patient care. Midwives were trained using role plays and simulation and given specific feedback. Field officer and DPHNs conducted follow-up visits with the midwives and observed the telemedicine care to support quality implementation. Figure 1 shows the recommended schedule of ANC visits.

### Intervention design

Initial discussions were held between UNFPA, DGNM, the Directorate General of Health Services and implementing partners about the program. Pandemic-related funding was sought and obtained from SIDA for implementation in the existing five program supported districts: Dhaka, Cox’s Bazar, Bandarban, Noakhali and Sunamganj. Districts were selected as part of the larger project based on vulnerability criteria, including high unmet need for family planning, neonatal mortality rates, maternal death (crude death rate), and low numbers of antenatal care visits and skilled birth attendant rates.

Due to reports of increasing GBV during pandemic lockdowns [4, 5], it was determined that GBV screening and referral should be incorporated into the service. An orientation session was held to sensitize key stakeholders comprising district public health officers, project mentoring and monitoring officers, nursing supervisors, midwives, and hospital statisticians who would be involved in implementation. The early discussions drew attention to the lack of an existing system for scheduling client visits. Prior to this, clients determined when they visited facilities for ANC based on providers’ suggestions; subsequent visit appointments were not recorded at facilities. Only a handful of facilities used ANC cards which are carried by the patients in which suggested days for return visits were documented. It was initially thought that clients would receive midwives’ mobile phone numbers and be invited to call if needed. While other telemedicine programs in Bangladesh are set up that way, later design discussions resulted in the determination that appointment scheduling, led by midwives, would ensure that all women, rather than just those initiating phone calls, would be followed up. This decision initiated a significant change in standard procedure. Version 1 of a scheduling tool was developed and in November 2020 a three-day training of trainers was held to begin roll out (Additional file 3).

The training brought together 90 midwives (20 in-person and 70 virtually) as well as nursing supervisors, and district officers who oversee midwifery, and was led by the DGNM with support from UNFPA. It provided detailed guidance on organizing and carrying out telemedicine services including both the above mentioned ANC and PNC guide, and a UNFPA supported Ministry of Health screening and referral guide for GBV survivors. The GBV protocol had four components: (1) observation of signs and symptoms, (2) probing questions to determine (a) if a woman can safely speak freely and (b) to create an opportunity for disclosure, (3) guidance for providing first line counseling and clinical services, and (4) making an appropriate referral.

A smart phone was provided to each facility for midwives to share with monthly charges paid to facilitate phone calls and data entry for reporting (see Fig. 3 for data flow). Using the visit schedule and checklists in the *Technical Brief for Maternity Services,* and standard GBV protocol, midwives were instructed to follow these steps:

- At the first onsite (face-to-face) visit, determine the gestational age.
- Based on the gestational age, decide when a phone call will be and schedule it within one month, one week or 2–5 days based on the gestational age.
- Write patient's name and phone number in the scheduling tool for the agreed upon day.
- Check the scheduling tool daily to identify who needs to receive a call.
- Phone calls to be performed on the day they appear on the scheduling tool.
- Midwives use the ANC/PNC checklists and GBV protocol to guide phone calls.
- At the end of each phone call, schedule the next on-site visit.

Though midwives were instructed to attempt to reach women multiple times as needed, there was no specific guidance on how many calls should be attempted before considering the visit a no show.

### Roll out

In December 2020, telemedicine services were introduced in 13 of 36 (36%) sub-district hospitals in the five districts. Initially, all 36 hospitals in the selected districts were to adopt the intervention. This was reduced to 13 that were deemed to have adequate midwife staffing, which was considered to be three or more midwives. At the facility level, nursing supervisors oversaw midwives’ adherence to the agreed guidelines for the telemedicine calls. A supervision system was also established with monthly visits from field officers funded through UNFPA and government public health nurses, both embedded within district health offices. During these visits, documentation of client visits and discussions on progress made were reviewed with the onsite midwives. Cross-district supervision comprised meetings that served as a platform for discussing challenges and lessons learned. In July 2021, once the other 23 hospitals had at least 3 midwives employed onsite, they adopted the intervention as well. An additional 200 midwives were trained as part of the expansion.

### Monitoring and reporting

**Fig. 2:**
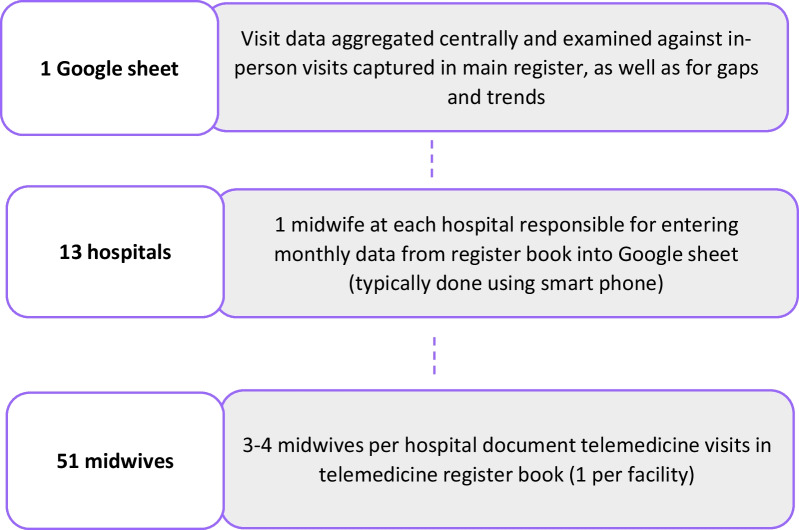
Data flow for reporting

Consideration was given to whether telemedicine visits should be recorded in the government register book that was used for onsite visits. Ultimately because this was a pilot and not yet part of the standard government system, telemedicine visits including both health and GBV notations were documented in a separate format from the onsite patient information. Telemedicine visits were put in an encrypted Google Sheet accessed via password protected devices that was part of a project package including the scheduling tool. This was a temporary measure until the government reporting system could be expanded to include telemedicine. The encrypted sheet captured whether a planned visit occurred, the ANC/PNC visit number, services provided (e.g., FP or GBV) as well as information from the visit. The data flow process is depicted in Fig. 2.

In-person visits were documented in the existing ANC register which was kept locked when not in use. Ongoing program monitoring revealed the need to redesign the scheduling tool to an easier to manage format. Version 2 of the tool, designed as a standard appointment book, was well received as there was more space for recording scheduled appointments (Additional file 4).

## Learnings

### Qualitative

Qualitative analysis identified nine themes (Table 1) and revealed both challenges and successes.

The earlier mentioned *Technical Brief on Maternity Services* guidance document did not include considerations for facilities that needed to introduce a scheduling system for patient visits. Yet, early conversations revealed that it was necessary to introduce one for midwives to keep track of when telemedicine visits for a given woman were due. This was a significant practice adjustment that had a learning curve. Once introduced, some midwives found it difficult to keep to the appointment schedule when the patient load was high, and managers talked about the importance of time management and organization. The following quotation from a midwife reflects that midwives felt busy handling both in-person and remote visits:

“The workload in both face-to-face and remote services is increasing due to the shortage of midwives in relation to the patient volume ratio in our hospital, resulting in an increase in workload. The quality of service will increase if the number of midwives increases.”

At the same time, service utilization increased. Another midwife shared:

“the ANC decreased during the [initial] COVID period, but gradually increased after the telemedicine program began. In addition, for PNC services, many mothers did not take the 2nd and 3rd PNC service. Now increased the number of PNC services through remote”.

Midwives also shared challenges reaching patients at times as some did not answer their phone or had given their husband’s or mother-in-law’s phone number. Women typically live in multigenerational households and are responsible for performing household chores through the day. It was thus not uncommon for them to have restrictions on their phone use placed by other family or household members.

Successes included midwives stating comfort with using a standard checklist for ANC and PNC and that they had adapted the checklist for onsite visits as well. As one service user described:

“the midwife reminded me of birth preparation, signs of maternal danger and important directions for nutrition.”

Midwives, managers, and women also noted that the program enabled relinking lost-to-follow-up women to services, and facilitated critical check-ins with high-risk pregnant mothers. One midwife stated:

“Telemedicine is making us closer to the pregnant women and pregnant women benefit from important messages through these phone calls. During the remote ANC sessions we answer the pregnant women’s queries and counsel them on side effects of drugs, COVID-19 prevention, family planning, and birth preparedness. Some of the pregnant women get curious after the call and come to the health facility to get checked for problems.”

A service user shared:

“In the ninth month of pregnancy, I noticed once that my baby was not moving. I called you midwife. Having heard everything, Apa (the midwife) asked me to go to the hospital and I went.”

Mangers stated that this service facilitates government initiatives for linking pregnant women to antenatal and maternity care. One manager stated:

“We received a directive from Dhaka to reach out to the communities and make sure all pregnant women come for services, the program helps us to do that.”

Mangers, midwives, and pregnant women acknowledged that the program prevented unnecessary hospital visits and improved the provider–client relationship. It also raised awareness about pregnancy risks and midwives’ services, and encouraged facility births. One service user stated that:

“The midwife reminded me about birth preparedness, maternal danger signs and important instructions for nutrition.”

Another stated:

“I was happy to receive the call from the midwife because I had been experiencing slight abdominal pain. I discussed all my problems and queries with the midwife and the midwife answered all patiently.”

Interviews with the midwives found that only eleven out of 14 interviewed midwives were screening for GBV and, perhaps related, both midwives and manager stated that women sometimes were reluctant to communicate about GBV when asked screening questions. One midwife stated:

“Most mothers don’t want to say that they suffer from gender-based violence but when they do we provide counseling to the victim women and refer them for further help based on their necessity.”

Interestingly, some midwives and managers felt women were more likely to disclose on the phone than in person. One manager stated:

“Women are more likely to share and get counseling on the mobile phone, as they usually feel shy to share through face-to-face discussion.”

### Quantitative

**Fig. 3:**
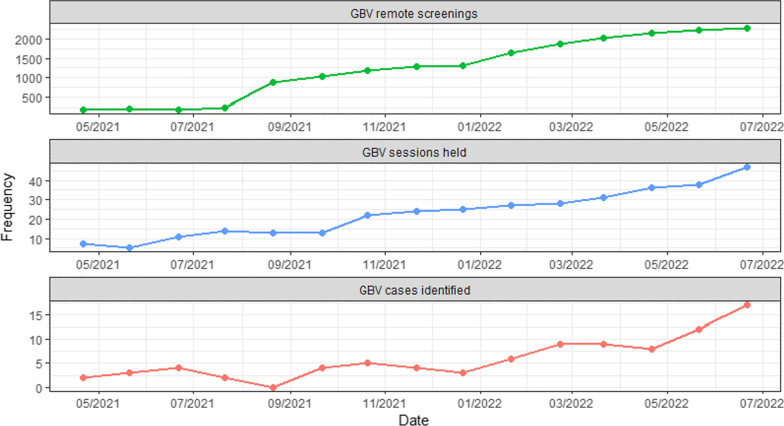
GBV screening, counseling and referral trends

While GBV service data showed that GBV screening occurred during 88% of telemedicine services (6917 screenings), only 230 women disclosed and received counseling, and only 33 agreed to be referred. In addition, as already mentioned, only 11 of the 14 midwives mentioned screening for GBV. One notable finding was that the number of women screened for GBV, as well as counseling related to disclosures, and referral increased significantly over time. Figure 3 shows the temporal trends for the frequency of each outcome variable of interest starting in April 2021. Data from the months of January through March of 2021 were excluded from the analysis due to irregularities in the data that were caused by midwives that were assigned to the project being reassigned to COVID hospitals during these months. Overall, these outcome variables showed an increase in frequency over time following the implementation of the telemedicine program.

Table 2 displays the results of the Poisson regression models. The model indicated a statistically significant increase over time in each of the outcome variables.

**Table 2:** Results of Poisson regression

	Dependent variable		
	GBV remote screenings	GBV sessions held	GBV cases identified
(Intercept)	5.739***	1.992***	0.299
Time	0.149***	0.124***	0.156***
Obs	15	15	15
Log Likelihood	− 712.959	− 39.138	− 30.202
AIC	1429.918	82.278	64.404

*p < 0.05, **p < 0.01, ***p < 0.001

**Fig. 4:**
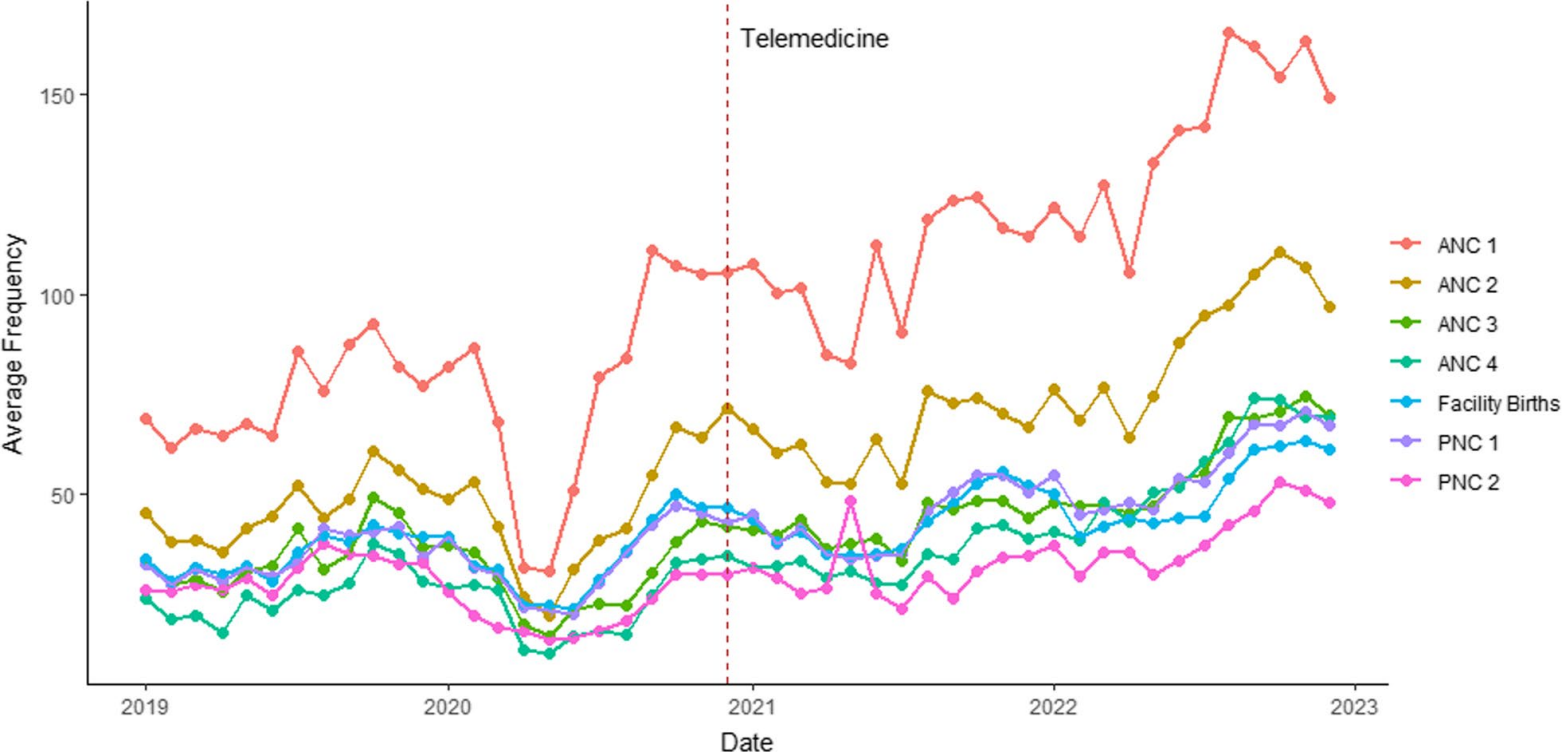
Time series plot of average frequencies across hospitals for the outcome variables ANC 1–4, facility births, PNC 1–2

**Fig. 5:**
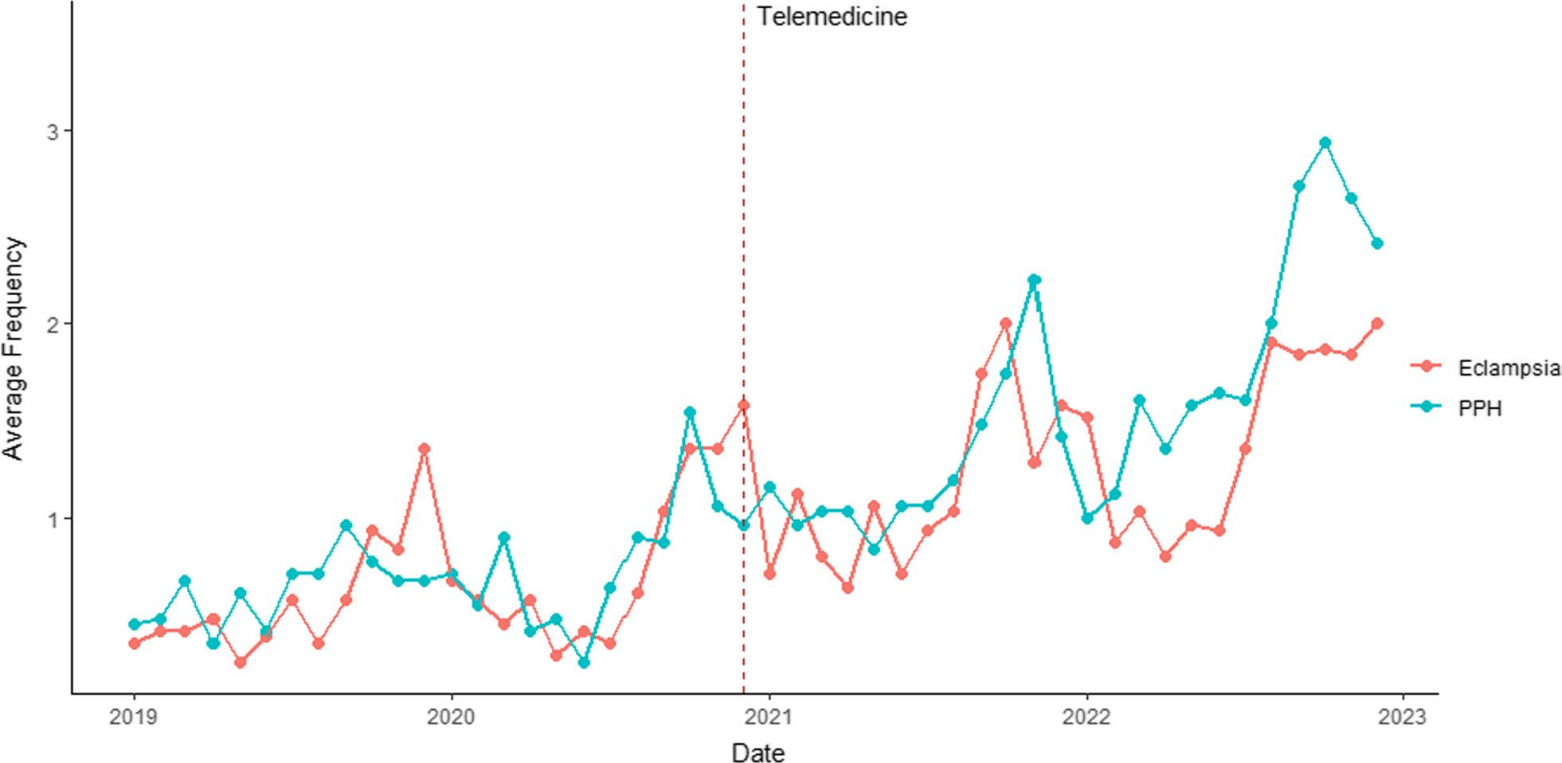
Time series plot of average frequencies across hospitals for the outcome variables eclampsia and PPH

Maternity care service use data however showed significant improvement in service utilization. Overall, remote visits comprised 17% and 15% of all ANC and PNC visits respectively. Service utilization data were compared to examine change over time from before the pandemic to after a year of telemedicine implementation. Total numbers of women seen at the 36 intervention facilities (both in-person and remotely) were looked at in 2019, 2020 and 2021. Figures 4 and 5 show the temporal trends for the frequency of each outcome variable of interest as they relate to the introduction of the telemedicine program in December 2020. Due to the much lower frequencies associated with eclampsia and PPH, these outcomes are shown separate from the other variables. Overall, the outcome variables exhibited an increase in slope following the implementation of the telemedicine program.

Table 3 presents the results of the multivariate test, demonstrating a significant overall effect of telemedicine on the trend of the outcome variables.

**Table 3:** Multivariate test

	F-test (approx.)	num DF	den DF	P-value
Time	3.1716	9	1476	< 0.001
Level	0.945	9	1476	0.4846
Trend	7.1724	9	1476	< 0.001

Table 4 displays the results of the interrupted time series analysis, presenting the coefficients for each outcome variable. The model indicated a statistically significant change in trend for ANC 1–4, PNC 1–2, and PPH following the introduction of telemedicine. The frequency of these outcomes exhibited an accelerated rate of increase after the implementation of the telemedicine program.

**Table 4:** Results of interrupted time series analysis

	Dependent variable								
	Eclampsia	ANC1	ANC2	ANC3	ANC4	PNC1	PNC2	PPH	Facility births
(Intercept)	0.330*	64.769***	41.719***	33.539***	23.309***	31.107***	30.702***	0.454***	31.269***
Time	0.026**	0.876	0.305	− 0.152	0.027	0.222	− 0.423	0.02*	0.274
Level	− 0.061	1.5	2.518	2.105	− 3.111	− 2.364	3.274	− 0.216	− 1.443
Trend	0.007	1.894*	1.612**	1.514***	1.818***	1.001**	1.238**	0.047***	0.555
Obs	1488	1488	1488	1488	1488	1488	1488	1488	1488
Adj. R^2^	0.045	0.062	0.064	0.053	0.097	0.047	0.016	0.091	0.029
F (df = 3; 1484)	24.215***	33.981***	35.002***	28.785***	54.505***	25.563***	8.806***	50.358***	15.954***

^*^p < 0.05, **p < 0.01, ***p < 0.001

These results show that (apart from facility births and eclampsia identification and management) service utilization surpassed not just the declines experienced at the onset of the COVID-19 pandemic, but also the pre-pandemic levels that reflected system-wide gaps. The data used for the analyses are available in Additional file 5.

### Mixed method analysis

Most respondents were positive with regard to the impact and potential of the program and that was reflected in the data which show large numbers of phone calls and improvements in health seeking behavior and service implementation for both routine and emergency care. Service users’ comments regarding the helpfulness of danger sign review is reflected in increases of women presenting with PPH and eclampsia seeking services at the health care facilities. Although some women did benefit from the GBV screening, the hesitance communicated both by some of the midwives by not adopting screening, and by their perception that women did not feel comfortable sharing, was reflected in the data in that the identified number of women needing counseling and referral was much less than what would be expected in the population of women receiving phone calls.

## Discussion

This study demonstrates that telemedicine, led by midwives, can be an effective approach for increasing maternity service utilization and life-saving care in a low-income setting, not just during a pandemic lockdown but to address ongoing gaps in care. While challenges existed—such as an initial need to institute a patient visit scheduling system, and that not all women were reachable by phone—overall there was a significant increase in women receiving both routine and emergency maternity care.

Concerns with the efficacy of screening for GBV arose, and thought is needed going forward to facilitate women’s comfort in receiving GBV support during ANC telemedicine visits. Notably the qualitative interviews revealed that some midwives and managers felt that women were more likely to disclose and receive counseling during telemedicine than during face-to-face visits. However, records were not kept of GBV disclosure during in-person visits. Also notable was that GBV screening, disclosure, and referral increased over time. This may be related to midwives feeling more comfortable asking about safety issues, as well as perhaps over time relationships were developed with the women that allowed for more trust, and thus disclosure.

Reticence in women to disclose GBV is also found in the literature]. Generally, women disclose more during provider interactions when they trust their provider, and when referral systems are available [10]. However, the literature focuses on in-person visits. More research is needed on barriers and facilitators to effective GBV screening specific to ANC provided during telemedicine.

Although a standardized tool was used for GBV screening, more consideration of effective tools based on the literature is needed going forward [25]. As was noted in this study, provider hesitance with GBV screening is known, with some research finding as few as 4.3% of providers screening despite it being protocol. Research is needed to identify how to support providers with screening, including identifying the most efficacious tool [26]. Adequate provider training, and supportive policies are found to facilitate implementation of GBV screening in ANC [27].

One notable aspect of the gaps in guidance for GBV screening during telemedicine is that although the importance of privacy and confidentiality within both GBV and health systems is well known, the steps for ensuring the level of privacy needed when addressing a case of GBV is not yet well established within health record keeping systems in many low- and middle-income countries [28, 29]. In these contexts, health data is commonly kept in registration books, as opposed to unique patient case sheets [30]. These register books, although locked when not in use, are accessible to all relevant health care providers and managers, as well as those reporting into health information systems. This is a critical and somewhat complicated concern as low resource settings may have gaps in what is needed to establish unique patient files [31]. There is an urgent need to strengthen systems in this regard. More literature is needed on barriers and facilitators for the integration of GBV services within antenatal care and the best methods to ensure protection for women who disclose.

Much of the existing literature on pandemic telemedicine describes the limitations inherent in models of remote maternity care, such as restricted mobile phone coverage in remote areas, inadequate training and support to implement at scale, lack of bonding and trust between patients and providers, and preferences among some women for in-person visits [32, 33]. In addition, quality of care is pointed out in particular as an area that needs more research [32, 34]. In addition to the physical aspects of ANC visits (e.g., checking blood pressure, weight and urine), routine supervision for quality improvement has not typically been part of programs.

This study was unique in that it identified that there was not system for scheduling patient visits in the facility. It is common in low-resource settings that unlike high resource systems, women are not given a date and time for their next appointment, but rather are told when to come back, and thus the onus is on the women. This more informal method is limited in that women can be easily lost to follow up, and is not effective in the context of telemedicine as the provider must initiate the care.

At the same time, studies have identified effective aspects of remote maternity care, such as bringing services to women who otherwise may not be able to access them due to distance from facility, lockdowns, or fear of contracting COVID-19. Few studies, however, describe telemedicine provided by midwives, and none were found that looked at GBV screening as part of health care services, or that examined the program's overall impact on service utilization. This study makes a novel contribution in that it documents the experience of midwife-led telemedicine in a low-income country, and it shows some success in identifying and referring women facing GBV. It also shows  notable success with increased maternity service utilization, a strong indicator of improved outcomes.

Another beneficial aspect of this telemedicine intervention in Bangladesh was that, by using midwives deployed through the national health system and relying on existing telephone technology rather than attempting to roll out a new telehealth platform, it was low-cost. This made it more sustainable than similar remote maternity care interventions implemented in South Asia [34, 35]. Cost is a major factor in maternal health care. A recent Lancet article announced a call to action for greater investment in maternal health to address the stagnating, and in some regions increasing, maternal mortality seen over the past decade [36]. Yet, while greater investment is urgently needed, midwife-led telemedicine provides a model for a low-cost intervention that can increase service utilization and link more women facing both GBV and obstetric emergencies in low-income countries to care. Future maternal and newborn telemedicine efforts should consider needs for basic program supports–such as mobile phones and phone credit as well as introduction of a standard scheduling system–for effective implementation. In addition, approaches for quality supervision in remote maternity care should be studied to integrate systems for continuous quality improvement.

## Conclusion

Remote maternity care and GBV screening and referral via telemedicine led by midwives was an effective way to reach more women with life-saving services in Bangladesh. Implementation should consider the need for instituting scheduling systems and sustained support for mobile phone usage by midwives. Future research is recommended on GBV screening during ANC and on care quality in maternity telemedicine to inform supervision and quality improvement strategies.

### Supplementary Information

**Additional file 1:** COVID-19 Technical Brief for Antenatal Care Services 2020 published by United Nations Population Fund.

**Additional file 2:** COVID-19 Technical Brief for Postnatal Care Services 2020 published by United Nations Population Fund.

**Additional file 3:** Version 1 of the scheduling tool used in the telemedicine intervention.

**Additional file 4:** Version 2 of the scheduling tool used in the telemedicine intervention.

**Additional file 5:** Service utilization dataset that the quantitative analysis drew from.

## Data Availability

Qualitative datasets used and/or analyzed during the current study are available from the corresponding author on reasonable request. Quantitative data analyzed during this study are included in supplementary information files.
