# Supplementary material for: Midwife-led pandemic telemedicine services for maternal health and gender-based violence screening in Bangladesh: an implementation research case study
Source: Reprod Health. 2023 Aug 29;20:128. doi: 10.1186/s12978-023-01674-0 (PMC10466754; doi:10.1186/s12978-023-01674-0)
Supplement: Supplementary file 1 — Additional file 1: COVID-19 Technical Brief for Antenatal Care Services 2020 published by United Nations Population Fund. [file 12978_2023_1674_MOESM1_ESM.pdf]

COVID-19
Technical Brief for Antenatal Care Services

April 2020

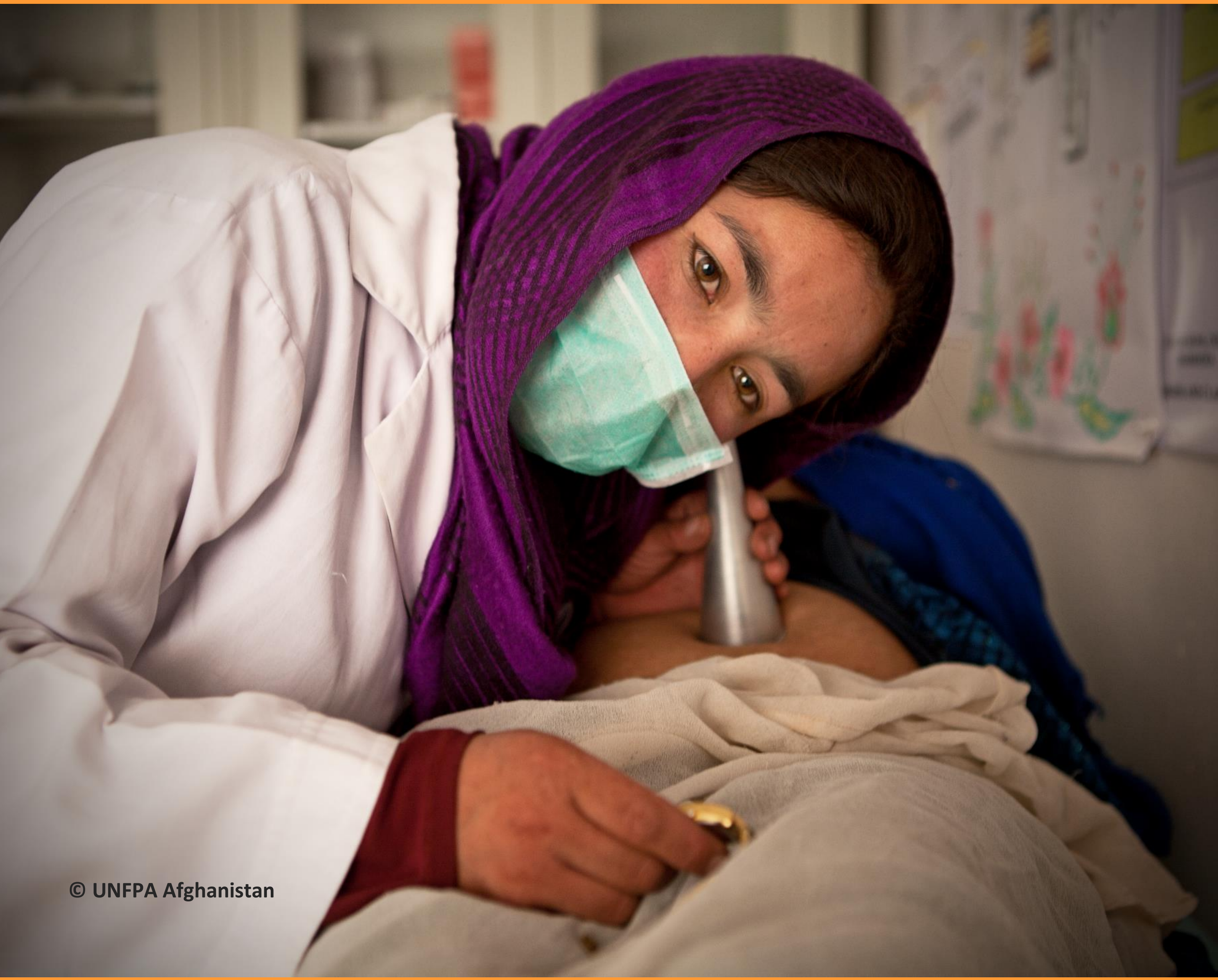

© UNFPA Afghanistan

Table of Contents

Introduction........ 3

Antenatal Care........ 4

Alternate Delivery of Antenatal Contacts........ 6

Alternate Delivery of Contact during COVID-19 Tables........ 7

- Table 1. Antenatal Contacts – Remote Contact available........ 7

Country-specific protocols that may require consideration........ 8

Remote Antenatal Contacts Checklist........ 9

- ANNEX 1: Face-to-Face Contact 1, and Remote Contacts 2 and 3........ 10
- ANNEX 2: Face-to-Face Contact 4, and Remote Contact 5........ 13
- ANNEX 3: Face-to-Face Contact 6, Remote Contact 7, Face-to-Face Contact 8........ 16

Reference List........ 19

Acknowledgments

This technical guidance was prepared by UNFPA in collaboration with the Burnet Institute, Australia. UNFPA wishes to express its sincere thanks to Ms Rachel Smith, Senior Midwifery Specialist, and Professor Caroline Homer, Co-Program Director, Maternal and Child Health, for their work in preparing and reviewing this document.

Antenatal Care during COVID-19

Introduction

It is anticipated that that COVID-19 (the disease caused by the novel coronavirus named SAR-CoV-2) will occur in most, if not all countries. A key fact about COVID-19 is that the vast majority of infections will result in very mild or no symptoms. Not everybody is at risk of severe disease. Persons of advancing age and those with existing respiratory, cardiac and/or metabolic disorders and immunodeficiencies are at higher risk of moderate to severe disease.

Limited data are available on COVID-19 in pregnancy, but the studies published to date do not show an increased risk of severe disease in late pregnancy or substantial risk to the newborn. Congenital infection has not been found, and the virus has not been detected in expelled products of conception. These findings are reassuring, and are quite different from other recent pandemics, like the 2009 H1N1 influenza A pandemic which resulted in more severe disease in pregnant women, or Zika virus which is teratogenic. Information on the impact of COVID-19 on early pregnancy outcomes remains unavailable at the time of writing. Non-pregnant women of childbearing age are also at low risk of severe disease [1].

The impact on acute care services in settings with under-resourced health systems is likely to be substantial. Maternity services should continue to be prioritized as an essential core health service, and other sexual and reproductive health care such as family planning, emergency contraception, treatment of sexually transmitted infections, and where legal safe abortion services, to the full extent of the law, also need to remain available as core health services.

Maternity care providers (including midwives and all other health care workers providing maternal and newborn care), whether based in health facilities or within the community, are essential health care workers and must be protected and prioritized to continue providing care to childbearing women and their babies. Deploying maternity care workers away from providing maternity care to work in public health or general medical areas during this pandemic is likely to increase poor maternal and newborn outcomes.

Maternity care providers have the right to full access for all personal protective equipment (PPE), sanitation and a safe and respectful working environment [2]. Maintaining a healthy workforce will ensure ongoing quality care for women and their newborns; without healthy midwives and other maternity care providers there will be limited care for women and newborns.

As part of COVID-19 Pandemic UNFPA Global Response Plan, the UNFPA response involves a 3-pronged approach for Maternity care:

1. Protect maternity care providers and the maternal health workforce
2. Provide safe and effective maternity care to women
3. Maintain and protect maternal health systems

Detailed practical recommendations across these 3 prongs for antenatal care, intrapartum and postnatal care have been outlined in: UNFPA COVID-19 Technical Brief for Maternity Services Interim Guidance, April 2020 [3].

This document serves as an adjunct to the UNFPA COVID-19 Technical Brief for Maternity Services (April 2020: https://www.unfpa.org/sites/default/files/resource-pdf/COVID-19_MNH_guidance_04.pdf) to provide interim guidance on providing phone based antenatal care (ANC) in the immediate clinical situation during COVID-19. These recommendations are provided as a resource for UNFPA staff based on a combination of WHO guidelines, good practice and expert advice based on the latest scientific evidence. The situation with COVID-19 is evolving rapidly and the guidance will continue to be updated if and when new evidence or information becomes available. An interim guidance on postnatal care is currently being developed and will be available shortly.

Antenatal Care

The overarching aim of this guidance is to ensure maternity care providers can deliver respectful and individualised antenatal care services that promote the safety of women, families and health professionals during the COVID-19 pandemic.

> *'All pregnant women, including those with confirmed or suspected COVID-19 infections, have the right to high quality care before, during and after childbirth. This includes antenatal, newborn, postnatal, intrapartum and mental health care.* (World Health Organization, 2020)'.

In the coming weeks and months maternity care providers will try to minimise direct patient contact in non-urgent situations in an attempt to minimise the spread of COVID-19 [1, 4, 5]. Adjustments to the standard antenatal care schedule may occur so that some antenatal appointments are conducted using telehealth[1], that is virtually by phone or video chat (remote contact)[2], to ensure that there is no disruption in service or breakdown in women's maternity care. Midwives and other key providers of antenatal care will need to use clinical judgement in deciding which women may be suitable for an alternate schedule of face to face care (contacts) that includes remote AN contacts. Primarily this will be women who have reliable mobile phone access are deemed low-risk – bearing in mind that risk status may change as pregnancy progresses so risk assessment must occur at every AN contact.

When it is necessary to physically examine women at an AN contact, the physical part of the examination will be undertaken respectfully but quickly to minimise time spent within the recommended 1-meter distancing [6].

Health services and clinics may:

- Triage and screen all women for symptoms of COVID-19 before entering the facility
- Limit the number of women attending clinics each day
- Change delivery modality scheduled AN contacts (after risk-assessment)
- Move AN clinics from hospital environments to the community and/or where possible recommend a route to the AN clinic that bypasses Emergency or designated Fever Clinics

[1] Telehealth involves the use of telecommunications and virtual technology to deliver health care outside of traditional health-care facilities.
[2] Referred to in this document as 'remote contact'

- Undertake non-physical assessments in open environments (outside)
- Limit attendance of support people such as partners/children (at AN contacts)
- Separate physical assessment from discussion/enquiry part of AN contact
- Provide a 'one-stop' contact meaning combining services such as USS, medication administration, blood and other tests at the same contact to prevent women having to return as frequently

[1, 2, 5-7].

The UNFPA COVID-19 Technical Brief for Maternity Services (April 2020) provides further recommendations about triage, exposure screening and organisation of facility based antenatal care services.

Regardless of where or how antenatal contact occurs, respectful maternity care must be at the forefront of the care provided. In these unprecedented times, women may be scared or anxious for themselves, their babies and their families. This fear and anxiety may be made worse by seeing their care providers in extensive personal protective equipment (PEE) as this can impact on simple actions such as seeing a kind smile. Health professions need to ensure every interaction with every woman is friendly, kind and respectful [8]. Where possible, continuity of midwifery care should be provided throughout the antenatal period, and indeed the birth and postpartum period. This is known to improve positive outcomes and will reduce the number of caregivers in contact with the woman and her birth partner [4, 9, 10].

The following document provides practical guidance on antenatal contacts undertaken remotely (phone/messaging application/telehealth). This guidance provides direction for services to continue to provide essential and respectful antenatal care during the COVID-19 pandemic. It is intended to support services in adjusting to a different way of delivering antenatal care but does not replace usual policies and protocols regarding antenatal care provision. Services should revert to the *WHO Recommendations on Antenatal Care for a Positive Pregnancy Experience* [10] guidance once the pandemic status is lifted.

Prior to commencing telehealth services:

- Develop a facility or health system strategy such as a health information management system, to introduce and monitor changes in AN contacts
- Provide staff with technology, training and systems to provide remote AN contacts including sufficient resources for midwives to undertake phone contacts (access to mobile phone, charger, pre-paid phone credit and sim card or money for purchasing phone credit)
- Obtain and document informed consent from the woman for remote AN contacts

Alternate Delivery of Antenatal Contacts

Unfortunately, data suggests that many countries are currently not meeting the WHO eight AN contact guidance and less than 65% of women receive at least four AN contacts [11]. Whilst this information does not change recommendations, it needs to be considered in light of potential disruption to usual services and may mean the resources, in already low-resource settings are diverted for the COVID response. This guidance, in no way supports disruption to, or reduction of sexual and reproductive services but understands that during the current COVID pandemic different ways of working may need to be considered to ensure all women, at the very least, have access to evidence-based antenatal care. Once the pandemic status is lifted it is vital that countries continue striving to provide the WHO recommended level of antenatal care.

Wherever possible **the current WHO schedule of eight AN contacts should be provided and maintained** [10, 12]. Where technology and services are available, some of these contacts may be a remote AN contact. The schedule below offers guidance on which contacts might best be undertaken face-to-face and which might suit a remote contact (Table 1.).

Regardless of type of contact ALL women need to have:

- Assessment for, and information on, possible COVID-19 symptoms*
    - If women report symptoms or contact with suspected/confirmed COVID provide country-specific information on mandatory self-isolation and advise phone contact or rescheduling where possible (if urgent need, follow Facility/Country recommendations for seeking care)
- Information on Danger Signs** in pregnancy and Birth Preparedness*** discussion [13]
- Ongoing pregnancy risk assessment – including emotional wellbeing and personal safety
    - If risk assessment identifies potential or actual complications more frequent contacts need to occur and these may need to be face-to-face
- Adequate documentation of care provision to ensure appropriate care planning

If necessary, services must develop a process for integrating remote contact documentation in women's hand-held records.

* COVID-19 Symptoms – fever, tiredness, dry cough, aches and pains, nasal congestion, runny nose, sore throat or diarrhea (World Health Organization, 2020)
**Danger signs include: Vaginal bleeding; Convulsions/fits; Severe headache and/or blurred vision; Fever and too weak to get out of bed; Severe abdominal pain; Fast or difficult breathing (World Health Organization, 2017)
*** Birth Preparedness planning includes knowing Danger Signs; planned birth place, skilled birth attendant and transport; identifying companion (World Health Organization, 2016)

Alternate Delivery of Contact during COVID-19 Tables

Table 1. Antenatal Contacts – Remote Contact available

Current WHO Recommended Antenatal Contacts	Alternate Modality of Antenatal Contact – where remote contact available (must have COVID-19 Symptoms, Danger Signs** and Birth Preparedness *** information)
**1 – 12 weeks**	Face to Face<br>Comprehensive history and plan for care<br>BP/ Blood tests<br>USS – where available<br>Initial risk assessment
**2 – 20 weeks**	**Remote contact** – including ongoing risk assessment
**3 – 26 weeks**	**Remote contact** – including ongoing risk assessment
**4 – 30 weeks**	Face-to-Face<br>BP/Blood tests and Abdominal Palpation including FHR.<br>Ongoing risk assessment
**5 – 34 weeks**	**Remote contact** - including ongoing risk assessment
**6 – 36 weeks**	Face-to-Face<br>BP/Blood tests and Abdominal Palpation including FHR.<br>Ongoing risk assessment<br>Birth planning
**7 – 38 weeks**	**Remote contact** – unless risk factors for hypertension in pregnancy or growth restriction identified previously
**8 – 40 weeks**	Face-to-Face<br>BP/Blood tests and Abdominal Palpation including FHR.<br>Ongoing risk assessment<br>Birth planning

* COVID-19 Symptoms – fever, tiredness, dry cough, aches and pains, nasal congestion, runny nose, sore throat or diarrhea (World Health Organization, 2020)
**Danger signs include: Vaginal bleeding; Convulsions/fits; Severe headache and/or blurred vision; Fever and too weak to get out of bed; Severe abdominal pain; Fast or difficult breathing (World Health Organization, 2017)
*** Birth Preparedness planning includes knowing Danger Signs; planned birth place, skilled birth attendant and transport; identifying companion (World Health Organization, 2016)

Country-specific protocols that may require consideration

Each country or practice setting may have protocols, policies and treatment regimens that need to be considered when altering standard AN contacts schedule and delivery modality. These may include but are not limited to:

- Regimes for, and supply of, Iron; Folic Acid; Calcium; and, other context-specific recommended supplementation
- Preventative measures/treatments such as:
  - anthelminthic prophylaxis and treatment
  - vaccination programs including tetanus toxoid
  - Malaria prophylaxis and treatment
  - antiretroviral therapy and HIV pre-exposure prophylaxis
  - routine disease and/or infection screening and treatment

Procedures for follow-up interventions, provision of supplies, and, compliance monitoring will need to be considered. Clear procedures are also required for documentation/record keeping and emergency referral processes.

Remote Antenatal Contacts Checklist

The following guidance is for remote antenatal contacts – standard practice should continue for all face-to-face visits. Where necessary, refer to local guidance on what should occur at usual antenatal contacts. This is not a comprehensive guide to content of antenatal visits – it is a guide to how remote visits might be structured.

Remote Antenatal Contacts Checklist
**ALL contacts regardless of method should include:**
**Respectful Maternity Care – includes:**<br>✓ Treating all women with dignity and respect<br>✓ Maintaining confidentiality and privacy<br>✓ Freedom from discrimination<br>✓ Supporting women's right to information and informed autonomous decision making
Suggested actions at every contact:<br>✓ Introduce yourself and greet the woman in a friendly manner<br>✓ **Assessment for possible COVID-19 symptoms (both woman and any support persons)** and refer to country/facility guidance or pathway for care if symptoms identified<br>✓ Enquire about the woman's general health and wellbeing<br>✓ Consider physical, social, emotional and cultural wellbeing<br>✓ Ask about pregnancy progress<br>✓ Undertake routine observation and assessment<br>✓ Explain all tests and procedures and obtain consent<br>✓ Review history and undertake ongoing assessment of risk factors<br>✓ Discuss danger signs<br>• Vaginal bleeding<br>• Convulsions/fits<br>• Severe headache and/or blurred vision<br>• Fever and too weak to get out of bed<br>• Severe abdominal pain<br>• Fast or difficult breathing<br>✓ Offer time for questions – take time to answer<br>✓ Provide gestation and pregnancy-specific information and education<br>✓ Undertake consultation and referral where necessary<br>✓ Discuss plan for emergency transport from the woman's home to a health facility if needed<br>✓ Plan for next AN contact and ongoing care<br>✓ Document assessments, discussions and plans for continued care

ANNEX 1: Face to Face Contact 1, and Remote Contacts 2 and 3

Initial Face-to-Face Contact 1 (12 weeks)

12 weeks<br>***(First trimester)***	**Standard first AN contact**<br>In addition to the standard first AN contact, assessments /activities need to ensure:<br>• Information about telehealth and schedule of AN contacts and obtain consent for phone/video calls. Confirm correct phone number for the woman and also a backup phone number<br>• Ensure the woman has a contact number for midwife/practitioner providing remote AN contact or hospital/health service contact<br>• Ensure woman enough iron, folic acid, calcium etc. to help avoid facility-based AN contact just to obtain supplies

Remote Contacts 2 and 3 (20 and 26 weeks)

Suggested actions at every contact:

- ✓ Introduce yourself and greet the woman in a friendly manner
- ✓ **Assessment for possible COVID-19 symptoms (both woman and any support persons)** and refer to country/facility guidance or pathway for care if symptoms identified
- ✓ Enquire about the woman's general health and wellbeing
- ✓ Consider physical, social, emotional and cultural wellbeing
- ✓ Ask about pregnancy progress
- ✓ Undertake routine observation and assessment
- ✓ Explain all tests and procedures and obtain consent
- ✓ Review history and undertake ongoing assessment of risk factors
- ✓ Discuss danger signs
  - Vaginal bleeding
  - Convulsions/fits
  - Severe headache and/or blurred vision
  - Fever and too weak to get out of bed
  - Severe abdominal pain
  - Fast or difficult breathing
- ✓ Offer time for questions – take time to answer
- ✓ Provide gestation and pregnancy-specific information and education
- ✓ Undertake consultation and referral where necessary
- ✓ Discuss plan for emergency transport from the woman's home to a health facility if needed
- ✓ Plan for next AN contact and ongoing care
- ✓ Document assessments, discussions and plans for continued care

20 and 26 weeks<br>***(Second trimester)***	Introduce self and friendly greeting<br>• Respectful Maternity Care	☐
	How are you feeling today?	☐
	Calculate and confirm gestation with woman	☐
	How is your pregnancy going so far?<br>• Discuss any results from previous AN contact(s)	☐

	Were any problems identified at your initial AN contact?<br>• Ongoing risk assessment	☐
	Do you feel your baby/self getting bigger?<br>• Maternal impression on growth	☐
	Tell me about how your baby moves?<br>• Discuss expected patterns of movement as pregnancy progresses	☐
	Are you eating and drinking well?<br>• Consider dietary advice	☐
	Are you taking any medications (Iron/parasite etc)?<br>• Discuss routine supplementation/medications	☐
	Are you having any trouble going to the toilet?<br>• Signs of UTI?<br>• Constipation?	☐
	Have you noticed any danger signs?<br>• Vaginal bleeding<br>• Convulsions/fits<br>• Severe headache and/or blurred vision<br>• Fever and too weak to get out of bed<br>• Severe abdominal pain<br>• Fast or difficult breathing<br>**If danger sign present – consult or refer** as per usual practice/policy	☐
	What would you do if you did have any danger signs?<br>• Start Birth Preparedness discussion<br>• Include where and how to seek help	☐
	Would you be able to get to the nearest health service? How?<br>• Access to transport<br>• Finances	☐
	Are you worrying a lot about anything?<br>• Emotional assessment	☐
	Are you having trouble sleeping?<br>• Emotional assessment	☐
	Do you feel safe at home?<br>• IPV/GBV assessment<br>• Provide information on services and contact number (where available)	☐
	Have you got some people who can provide you with support or help you if you need help?<br>• Emotional support<br>• Safety planning	☐
	Consider Health Promotion education<br>• Breastfeeding and early skin-to-skin<br>• Family planning and Birth Spacing<br>• Preventative treatments used in context of practice	☐
	Depending on schedule of contacts:<br>• Make next appointment	☐

	Do you have any other questions? Or Is there anything you want to talk about?	☐
	Remind the woman of importance of antenatal care, keeping her next AN contact and the process to follow if she has concerns regarding pregnancy or any danger signs.	☐

Consider:

- ✓ Is this woman due for any routine testing – can it be delayed until the next face-to-face AN contact?
- ✓ What information or education does this woman need at this gestation?
  - Discuss signs of preterm labour and what to do if she thinks labour is starting
  - Discuss fetal movements and what to do if she notices a change in the movement pattern
- ✓ Have you identified any new risk factors? If so,
- ✓ Does this woman need a face-to-face AN contact?
- ✓ Does this woman know the referral pathway for accessing hospital services during COVID-19 (i.e.: Do pregnant women need to be seen in a different location to usual at the hospital? Where do they go for triage and initial exposure risk screen etc?)
- ✓ How/where will you document this contact?
- ✓ Document date and details of previous phone contact in woman's hand held record

ANNEX 2: Face to Face Contact 4, and Remote Contact 5		
**Face-to-Face Contact 4 (at approximately 30 weeks)**		
30 weeks (Third trimester)	Standard subsequent AN contact	
**Remote Contact 5 (34 Weeks)**		

Suggested actions at every contact:

- ✓ Introduce yourself and greet the woman in a friendly manner
- ✓ **Assessment for possible COVID-19 symptoms (both woman and any support persons)** and refer to country/facility guidance or pathway for care if symptoms identified
- ✓ Enquire about the woman's general health and wellbeing
- ✓ Consider physical, social, emotional and cultural wellbeing
- ✓ Ask about pregnancy progress
- ✓ Undertake routine observation and assessment
- ✓ Explain all tests and procedures and obtain consent
- ✓ Review history and undertake ongoing assessment of risk factors
- ✓ Discuss danger signs
  - Vaginal bleeding
  - Convulsions/fits
  - Severe headache and/or blurred vision
  - Fever and too weak to get out of bed
  - Severe abdominal pain
  - Fast or difficult breathing
- ✓ Offer time for questions – take time to answer
- ✓ Provide gestation and pregnancy-specific information and education
- ✓ Undertake consultation and referral where necessary
- ✓ Discuss plan for emergency transport from the woman's home to a health facility if needed
- ✓ Plan for next AN contact and ongoing care
- ✓ Document assessments, discussions and plans for continued care

34 weeks (Third trimester)	Introduce self and friendly greeting<br>• Respectful Maternity Care	☐
	How are you feeling today?	☐
	Calculate and confirm gestation with woman	☐
	How is your pregnancy going so far? Or How have you been going since the last AN contact?<br>• Discuss any results from previous AN contact(s)	☐
	Have you had any problems identified that might affect your pregnancy?<br>• Ongoing risk assessment<br>• May require prompting – bleeding, diabetes, blood pressure etc	☐
	Was your baby's growth measured at your last AN contact – did the midwife have any concerns?<br>Do you feel your baby has grown since then?<br>• Maternal impression on growth	☐

	Tell me about how your baby moves?<br>• Discuss normal patterns of movement as pregnancy progresses<br>• Provide information on what to do if she feels her baby is moving less or differently to usual	☐
	Are you eating and drinking well?<br>Do you feel like you are gaining weight?<br>• Consider dietary advice	☐
	Are you taking any medications (Iron/parasite etc)?<br>• Discuss any routine supplementation/medications	☐
	Are you having any trouble going to the toilet?<br>• Signs of UTI?<br>• Constipation?	☐
	Have you noticed any danger signs?<br>• Vaginal bleeding<br>• Convulsions/fits<br>• Severe headache and/or blurred vision<br>• Fever and too weak to get out of bed<br>• Severe abdominal pain<br>• Fast or difficult breathing<br>**If danger sign present – consult or refer** as per usual practice/policy	☐
	What would you do if you did have any danger signs?<br>• Start Birth Preparedness discussion<br>• Include where and how to seek help	☐
	Tell me some signs that might indicate your labour is starting?<br>• Discuss preterm labour and when to seek help<br>• Discuss spontaneous rupture of membranes and when to seek help	☐
	Who will be your companion in labour?<br>• Discuss importance of companion in labour	☐
	Would you be able to get to the nearest health service? How?<br>• Access to transport<br>• Finances	☐
	Are you worrying a lot about anything?<br>• Emotional assessment	☐
	Are you having trouble sleeping?<br>• Emotional assessment	☐
	Do you feel safe at home?<br>• IPV/GBV assessment	☐
	Have you got some people who can provide you with support or help you if you need help?<br>• Emotional support<br>• Safety planning	☐
	Consider Health Promotion education<br>• Breastfeeding and early skin-to-skin<br>• Family planning and Birth Spacing	☐

	• Preventative treatments used in context of practice	
	Depending on schedule of contacts:<br>• Make next appointment	☐
	Do you have any other questions?	☐
	Remind the woman of importance of antenatal care, keeping her next AN contact and the process to follow if she has concerns regarding pregnancy, onset of labour or any danger signs.	☐

Consider:

- ✓ Is this woman due for any routine testing – can it be delayed until the next face-to-face AN contact?
- ✓ What information or education does this woman need at this gestation?
  - Discuss signs of preterm labour and what to do if she thinks labour is starting
  - Discuss fetal movements and what to do if she notices a change in the movement pattern
  - Discuss birth preparedness
- ✓ Have you identified any new risk factors? If so,
- ✓ Does this woman need a face-to-face AN contact?
- ✓ How/where will you document this contact?
- ✓ Document date and details of previous phone contact in woman's hand held record

Annex 3: Face to Face Contact 6, Remote Contact 7, Face to Face Contact 8

Face-to-Face Contact 6 (at approximately 36 weeks)	
36 weeks ***(Third trimester)***	Standard subsequent AN contact

Remote Contact 7 (38 Weeks)

Suggested actions at every contact:

- ✓ Introduce yourself and greet the woman in a friendly manner
- ✓ **Assessment for possible COVID-19 symptoms (both woman and any support persons)** and refer to country/facility guidance or pathway for care if symptoms identified
- ✓ Enquire about the woman's general health and wellbeing
- ✓ Consider physical, social, emotional and cultural wellbeing
- ✓ Ask about pregnancy progress
- ✓ Undertake routine observation and assessment
- ✓ Explain all tests and procedures and obtain consent
- ✓ Review history and undertake ongoing assessment of risk factors
- ✓ Discuss danger signs
  - Vaginal bleeding
  - Convulsions/fits
  - Severe headache and/or blurred vision
  - Fever and too weak to get out of bed
  - Severe abdominal pain
  - Fast or difficult breathing
- ✓ Offer time for questions – take time to answer
- ✓ Provide gestation and pregnancy-specific information and education
- ✓ Undertake consultation and referral where necessary
- ✓ Discuss plan for emergency transport from the woman's home to a health facility if needed
- ✓ Plan for next AN contact and ongoing care
- ✓ Document assessments, discussions and plans for continued care

38 weeks ***(Late third trimester)***	Introduce self and friendly greeting • Respectful Maternity Care	☐
	How are you feeling today?	☐
	Calculate and confirm gestation with woman	☐
	How is your pregnancy going so far? Or How have you been going since the last AN contact? • Discuss any results from previous AN contact(s)	☐
	Have you had any problems identified that might affect your pregnancy? • Ongoing risk assessment • May require prompting – bleeding, diabetes, blood pressure etc	☐
	Was your baby's growth measured at your last AN contact – did the midwife have any concerns? Do you feel your baby has grown since then? • Maternal impression on growth	☐

	Tell me about how your baby moves?<br>• Discuss normal patterns of movement as pregnancy progresses<br>• Provide information on what to do if she feels her baby is moving less or differently to usual	☐
	Are you eating and drinking well?<br>Do you feel like you are gaining weight?<br>• Consider dietary advice	☐
	Are you taking any medications (Iron/parasite etc)?<br>• Discuss any routine supplementation/medications	☐
	Are you having any trouble going to the toilet?<br>• Signs of UTI?<br>• Constipation?	☐
	Have you noticed any danger signs?<br>• Vaginal bleeding<br>• Convulsions/fits<br>• Severe headache and/or blurred vision<br>• Fever and too weak to get out of bed<br>• Severe abdominal pain<br>• Fast or difficult breathing<br>**If danger sign present – consult or refer** as per usual practice/policy	☐
	What would you do if you did have any danger signs?<br>• Birth Preparedness discussion<br>• Include where and how to seek help	☐
	Tell me some signs that might indicate your labour is starting<br>• Discuss spontaneous rupture of membranes and when to seek help	☐
	Who will be your companion in labour?<br>• Discuss importance of companion in labour (WHO recommendation) and the need to ensure the companion is well and free of COVID symptoms - discuss local restrictions if present<br>• Discuss the need to plan for an alternate birth companion	☐
	Would you be able to get to the nearest health service? How?<br>• Access to transport<br>• Finances	☐
	Are you worrying a lot about anything?<br>• Emotional assessment	☐
	Are you having trouble sleeping?<br>• Emotional assessment	☐
	Do you feel safe at home?<br>• IPV/GBV assessment	☐
	Have you got some people who can provide you with support or help you if you need help?<br>• Emotional support<br>• Safety planning	☐

<table>
<tr><td rowspan="4"></td><td>Consider Health Promotion education
Breastfeeding and early skin-to-skin
Family planning and Birth Spacing
Preventative treatments used in context of practice</td><td>☐</td></tr>
<tr><td>Make next appointment</td><td>☐</td></tr>
<tr><td>Do you have any other questions?</td><td>☐</td></tr>
<tr><td>Remind the woman of importance of antenatal care, keeping her next AN contact and the process to follow if she has concerns regarding pregnancy, onset of labour or any danger signs.</td><td>☐</td></tr>
<tr><td colspan="3">Consider:
✓ Is this woman due for any routine testing – can it be delayed until the next face-to-face AN contact?
✓ What information or education does this woman need at this gestation?
Discuss signs of labour and what to do if she thinks labour is starting
Discuss importance of a skilled birth attendant for labour and birth
Discuss fetal movements and what to do if she notices a change in the movement pattern
Discuss birth preparedness
Discuss what the woman needs to bring with her when she comes in for the birth
Discuss breastfeeding and immediate skin-to-skin contact
Discuss potential for early discharge / modified schedule of in-person PN AN contacts in context of COVID-19
✓ Have you identified any new risk factors? If so,
✓ Does this woman need a face-to-face AN contact?
✓ How/where will you document this contact?
✓ Document date and details of previous phone contact in woman’s hand held record</td></tr>
<tr><td colspan="3">Face-to-Face Contact (39-40 weeks)</td></tr>
<tr><td>39-40 weeks
(Late third trimester)</td><td colspan="2">Standard subsequent AN contact
If further AN contacts required beyond 40 weeks, these must be face-to-face</td></tr>
</table>

Reference List

1. RCOG & RCM, *Coronavirus (COVID-19) Infection in Pregnancy. Information for health care professionals. Version 5, published Saturday 28th March 2020*. 2020, Royal College of Obstetricians and Gynaecologists and Royal College of Midwives: London.
2. International Confederation of Midwives (ICM), *Women's Rights in Childbirth Must be Upheld During the Coronavirus Pandemic*. 2020, International Confederation of Midwives: The Hague.
3. UNFPA, *Coronavirus Disease (COVID-19) Preparedness and Response UNFPA Interim Technical Brief* 2020, UNFPA: New York.
4. RCOG & RCM, *Guidance for antenatal and postnatal services in the evolving coronovirus (COVID-19) pandemic: Information for health professionals*. 2020, Royal College of Obstetricians & Gynaecologists and The Royal College of Midwives.
5. Royal Australian and New Zealand College of Obstetricians and Gyneacologists (RANZCOG), *Coronavirus Disease (COVID-19) in Pregnancy: A guide for resource-limited environments*. 2020, Royal Australian and New Zealand College of Obstetricians and Gyneacologists: Melbourne, Australia.
6. World Health Organization. *Q&A on COVID-19, pregnancy, childbirth and breastfeeding*. 2020 [cited 2020 1st April]; Available from: https://www.who.int/news-room/q-a-detail/q-a-on-covid-19-pregnancy-childbirth-and-breastfeeding.
7. Queensland Health, *Queensland Clinical Guidelines Perinatal care of suspected or confirmed COVID-19 pregnant women. Guideline No. MN20.63-V1-R25.* . 2020, State of Queensland (Queensland Health) Queensland, Australia.
8. The White Ribbon Alliance. *Respectful Maternity Care Charter*. 2011 [cited 2020 03/04/2020]; Available from: https://www.whiteribbonalliance.org/respectful-maternity-care-charter/.
9. Sandall, J., et al., *Midwife-led continuity models versus other models of care for childbearing women.* Cochrane Database of Systematic Reviews, 2016(4).
10. World Health Organization, *WHO recommendations on antenatal care for a positive pregnancy experience*. 2016, World Health Organization: Geneva.
11. UNICEF. *Antenatal Care: Percentage of women aged 15–49 attended by any provider at least four times during pregnancy (ANC4), trend estimates for 2006-2012, 2013-2018* 2019 [cited 2020 7 April]; Available from: https://data.unicef.org/topic/maternal-health/antenatal-care/.
12. World Health Organization. *WHO recommendation on antenatal care contact schedules*. 2018 [cited 2020 7 April]; Available from: https://extranet.who.int/rhl/topics/improving-health-system-performance/who-recommendation-antenatal-care-contact-schedules.
13. World Health Organization. (2017). *Managing complications in pregnancy and childbirth: a guide for midwives and doctors - Second Edition*. Retrieved from Geneva: https://apps.who.int/iris/bitstream/handle/10665/255760/9789241565493-eng.pdf?sequence=1
