# Supplementary material for: Midwife-led pandemic telemedicine services for maternal health and gender-based violence screening in Bangladesh: an implementation research case study
Source: Reprod Health. 2023 Aug 29;20:128. doi: 10.1186/s12978-023-01674-0 (PMC10466754; doi:10.1186/s12978-023-01674-0)
Supplement: Supplementary file 2 — Additional file 2: COVID-19 Technical Brief for Postnatal Care Services 2020 published by United Nations Population Fund. [file 12978_2023_1674_MOESM2_ESM.pdf]

COVID-19
Technical Brief
for Postnatal Care Services

April 2020

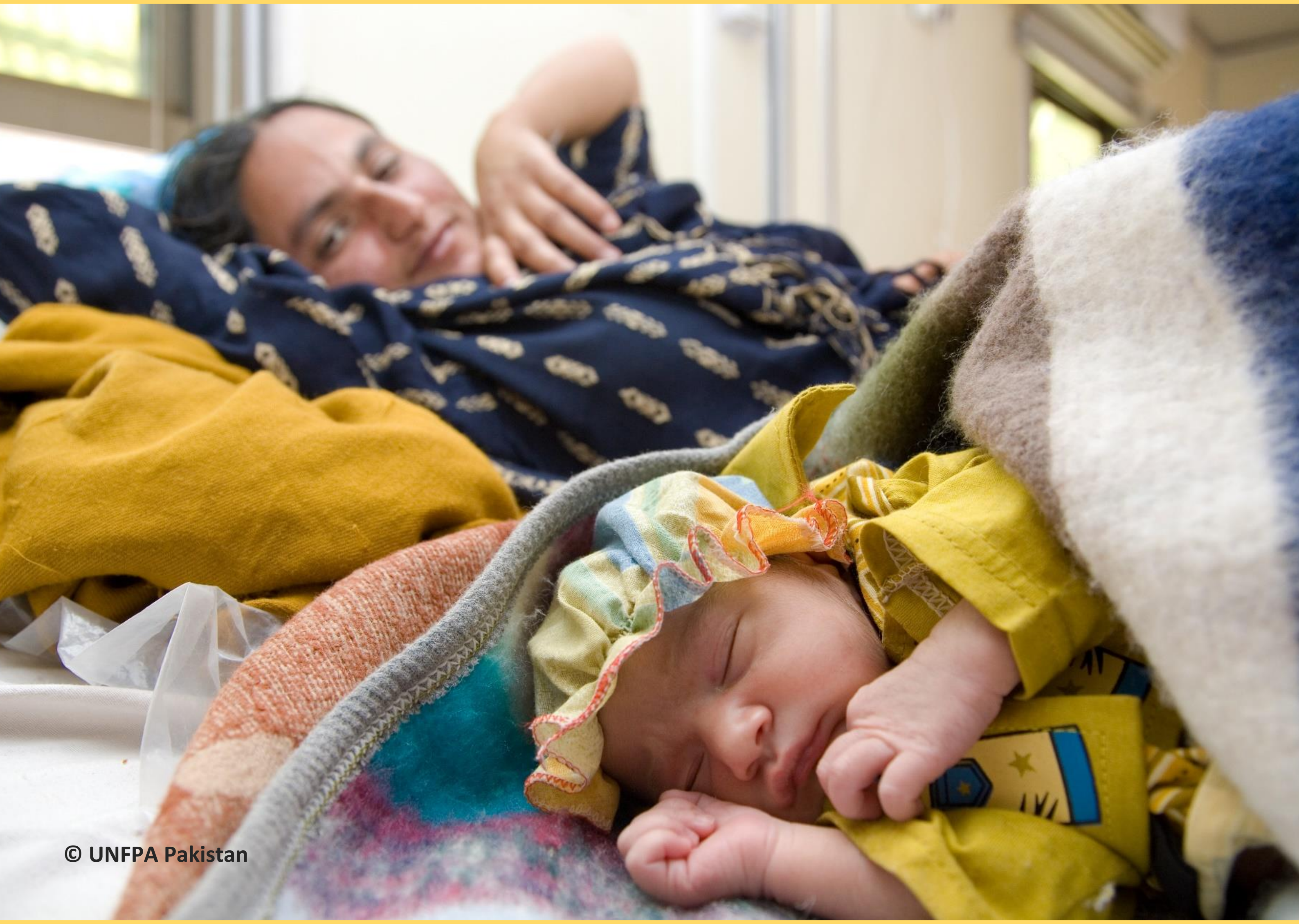
© UNFPA Pakistan

Table of Contents

Introduction…… 3

Postnatal Care…… 4

Alternate Delivery of Postnatal Contacts…… 6

Table 1. Table 1. Postnatal Contacts – Remote contact available…… 7

Remote Postnatal Contacts Checklist…… 8

Remote Postnatal Contacts…… 9

Reference List…… 14

Acknowledgments

This technical guidance was prepared by UNFPA in collaboration with the Burnet Institute, Australia. UNFPA wishes to express its sincere thanks to Ms Rachel Smith, Senior Midwifery Specialist, and Professor Caroline Homer, Co-Program Director, Maternal and Child Health, for their work in preparing and reviewing this document.

Postnatal Care during COVID-19

Introduction

It is anticipated that COVID-19 (the disease caused by the novel coronavirus named SAR-CoV-2) will occur in most, if not all countries. A key fact about COVID-19 is that the vast majority of infections will result in very mild or no symptoms. Not everybody is at risk of severe disease. Persons of advancing age and those with existing respiratory, cardiac and/or metabolic disorders and immunodeficiencies are at higher risk of moderate to severe disease.

Limited data are available on COVID-19 in pregnancy, but the studies published to date do not show an increased risk of severe disease in late pregnancy or substantial risk to the newborn. Information on the impact of COVID-19 on early pregnancy outcomes remains unavailable at the time of writing. Non-pregnant women of childbearing age are also at low risk of severe disease [1, 2].

The impact on acute care services in settings with under-resourced health systems is likely to be substantial. **Maternity services should continue to be prioritized as an essential core health service, and other sexual and reproductive health care such as family planning, emergency contraception, treatment of sexually transmitted infections, access to gender based violence support services and where legal safe abortion services, to the full extent of the law, also need to remain available as core health services.** [3]

Maternity care providers (including midwives and all other health care workers providing maternal and newborn care), whether based in health facilities or within the community, are essential health care workers and must be protected and prioritized to continue providing care to childbearing women and their babies. Deploying maternity care workers away from providing maternity care to work in public health or general medical areas during this pandemic is likely to increase poor maternal and newborn outcomes.

Maternity care providers have the right to full access for all personal protective equipment (PPE), sanitation and a safe and respectful working environment [4]. Maintaining a healthy workforce will ensure ongoing quality care for women and their newborns; without healthy midwives and other maternity care providers there will be limited care for women and newborns.

As part of the COVID-19 pandemic, the UNFPA response involves a 3-pronged approach:

1. Protect maternity care providers and the maternal health workforce
2. Provide safe and effective maternity care to women
3. Maintain and protect maternal health systems

Detailed practical recommendations across these 3 prongs for antenatal care, intrapartum and postnatal care have been outlined in: UNFPA COVID-19 Technical Brief for Maternity Services Interim Guidance, April 2020 (UNFPA):https://www.unfpa.org/sites/default/files/resource-pdf/COVID-19_MNH_guidance_04.pdf) [3].

This document serves as an adjunct to the Technical Brief to provide interim guidance on providing telehealth postnatal care (PNC) in the immediate clinical situation. These recommendations are provided as a resource for UNFPA staff, maternity care providers and other agencies, based on a combination of WHO and other maternity-based organizations' guidelines, good practice and expert advice based on the latest scientific evidence. The situation with COVID-19 is evolving rapidly and the guidance will continue to be updated when new evidence or information becomes available.

Postnatal Care

The overarching aim of this guidance is to ensure maternity care providers can deliver respectful and individualised care services that promote the safety of women, babies, families and health professionals during the COVID-19 pandemic.

'All pregnant women, including those with confirmed or suspected COVID-19 infections, have the right to high quality care before, during and after childbirth. This includes antenatal, newborn, postnatal, intrapartum and mental health care. [5].

In the coming weeks and months maternity care providers will try to minimise inpatient length of stay and direct patient contact in non-urgent situations in an attempt to minimise the spread of COVID-19 [1, 5]. As a result, standard health facility postnatal care routines and follow-up community contacts may change. Well women and their babies may be discharged from health services earlier depending on changes to local policies, and some postnatal contacts may be undertaken using telehealth[1], that is virtually by phone or video chat (remote contact)[2], to ensure that there is no disruption in service or breakdown in women's maternity care. **Some contacts with the health facility might be replaced by home visits by appropriately trained health care workers.** Midwives and other key providers of postnatal care will need to use clinical judgement in deciding which women may be suitable for an alternate care pathway involving early discharge and some remote contacts. Primarily this will be women who have reliable mobile phone access and are deemed to be at low-risk of complications

When it is necessary to physically examine women during a contact, the physical part of the examination will be undertaken respectfully but quickly to minimise time spent within the recommended 1-meter distancing [5].

Health services and clinics may:

✓ Triage and screen all women and companions for signs and symptoms of COVID-19[3] before entering or leaving the facility

(See UNFPA Technical Brief: Maternity Services April 2020 for further recommendations about triage, exposure screening and organisation of facility based and remote antenatal/ postnatal care services) [3].

---

[1] Telehealth involves the use of telecommunications and virtual technology to deliver health care outside of traditional health-care facilities.
[2] Referred to in this document as 'remote contact'
[3] COVID-19 Symptoms – fever, tiredness, dry cough, aches and pains, nasal congestion, runny nose, sore throat or diarrhoea

- ✓ Restrict attendance for PNC visits to include only the women, an asymptomatic companion of choice and the maternity care provider. Wherever possible, children, additional family members and companions should not accompany the women into the clinic visit.
- ✓ Change delivery modality for scheduled postnatal contacts (after risk-assessment)
- ✓ Separate physical assessment from discussion/enquiry part of postnatal contact [1, 4, 6, 7]

Regardless of where or how postnatal contact occurs, respectful maternity care must be at the forefront of the care provided [4, 7]. In these unprecedented times, women may be scared or anxious for themselves, their babies and their families. This fear and anxiety may be made worse by seeing their care providers in extensive personal protective equipment (PPE) as this can impact on simple actions such as seeing a kind smile. Health professions need to ensure every interaction with every woman is friendly, kind and respectful.

Where possible, continuity of midwifery care should be provided throughout the postpartum period. This is known to improve positive outcomes and will reduce the number of caregivers in contact with the woman and her baby [3, 8].

The following document provides practical guidance on postnatal contacts undertaken remotely (phone/messaging application/telehealth). This guidance provides direction for services to continue to provide essential and respectful postnatal care during the COVID-19 pandemic. It is intended to support services in adjusting to a different way of delivering postnatal care **but does not replace** usual policies and protocols regarding postnatal care provision. Services should revert to the *WHO recommendations on postnatal care of the mother and newborn* guidance once the pandemic status is lifted [9].

Prior to commencing telehealth services:

- ✓ Develop a facility or health system strategy such as a health information management system, to introduce and monitor changes in postnatal contacts
- ✓ Provide staff with technology, training and systems to provide remote postnatal contacts including sufficient resources for midwives to undertake phone contacts (access to mobile phone, charger, pre-paid phone credit and sim card or money for purchasing phone credit)
- ✓ Obtain and document informed consent from the woman for remote postnatal contacts and ensure the woman is able to contact the health service if any concerns

Alternate Delivery of Contact during COVID-19

Postnatal care should be individualized in accordance with the woman and newborn needs. The WHO minimum recommended number of postnatal contacts is four [9]. Remote contact can be suitable for women and newborn who are considered low risk. When determining if women are suitable for remote contact, the maternity care provider needs to consider support available for the women and her parity – for example a low-risk, multiparous woman who has a history of successful breastfeeding may be suited to more remote contacts than a primiparous woman with minimal home support.

Face-to-face contact should be prioritized for women and/or newborn who have:

- ✓ Known or are at risk of social and emotional vulnerabilities, including gender-based violence and mental health issues
- ✓ Complicated or operative births
- ✓ Prematurity/low-birth weight
- ✓ Other maternal or newborn complexities. This may include issues with infant feeding that can't be adequately assessed remotely [1]

At each contact, regardless of the type, ALL women and newborn need to have:

- ✓ Assessment for, and information on, possible COVID-19 symptoms*
- ✓ If women report symptoms or contact with suspected/confirmed COVID provide country-specific information on mandatory self-isolation and advise phone contact or rescheduling where possible (if urgent need, follow Facility/Country recommendations for seeking care)
- ✓ Newborn Danger Signs** and Maternal Complications*** information and discussion
- ✓ Ongoing risk assessment – including emotional wellbeing and personal safety
- ✓ If risk assessment identifies potential or actual complications more frequent contacts need to occur and these may need to be face-to-face
- ✓ Adequate documentation of care provision to ensure appropriate care planning

If necessary, services must develop a process for integrating remote contact documentation in women's hand-held records.

Table 1. Postnatal Contacts – Remote contact available

<table>
<tr><th>Current WHO 2013 Postnatal Minimum Contact Recommendations [9]</th><th colspan="2">Alternate modality of postnatal contact – where remote contact available (must have COVID-19 symptoms*, Newborn Danger Signs** and Maternal Complications*** information and education)</th></tr>
<tr><td>Birth – 24 hours</td><td colspan="2"><u>Face-to-Face</u>
Assess infective risk and ensure recommended PPE worn
Routine face-to-face assessment and pre discharge checklist**** if applicable. Ensure provision of adequate routine and prescribed medication to avoid unnecessary visits to health services
Facility birth – discharge according to current, local guidelines. Ensure that women who are being discharged earlier than usual can be well supported at home and there are systems in place for ongoing home based and/or telephone support by a maternity care provider
Birth at Home – should have face-to-face assessment by Skilled Birth Attendant within 24 hours</td></tr>
<tr><td>Day 3 (48-72 hours)</td><td><u>Remote Contact</u>
Includes ongoing risk assessment</td><td rowspan="3">Modality can be swapped depending on individual woman's need</td></tr>
<tr><td>Week 2 (7-14 days)</td><td><u>Face-to-Face Contact</u>
Assess infective risk and ensure recommended PPE worn
Includes ongoing risk assessment</td></tr>
<tr><td>Week 6 (~42 days)</td><td><u>Remote Contact</u>
Includes ongoing risk assessment
Ensure contraceptive needs have been discussed and addressed</td></tr>
</table>

* COVID-19 Symptoms – fever, tiredness, dry cough, aches and pains, nasal congestion, runny nose, sore throat or diarrhoea [5].

** Newborn Danger Signs include: stopped feeding well; convulsions; fast breathing (rate≥60/min); severe chest indrawing; no spontaneous movement; fever (temp ≥37.5); low body temperature (temp <35.5); any jaundice in first 24hrs or yellow palms/soles at any age [9].

***Signs or symptoms of postpartum haemorrhage; pre-eclampsia/eclampsia; infection; and, thromboembolism

**** If available, use existing or consider introducing e.g. https://www.healthynewbornnetwork.org/hnn-content/uploads/PNC-Checklist_Asia-1.pdf [10]

This table is based on the minimum recommended number of postnatal visits. Many women would benefit from more intensive support in the postnatal period and this may include more remote or face-to-face contacts. The number of contacts should be based on each woman's individual needs.

For face-to-face contacts ensure appropriate PPE as per WHO guidelines [11, 12]. For further details see: https://apps.who.int/iris/bitstream/handle/10665/331498/WHO-2019-nCoV-IPCPPE_use-2020.2-eng.pdf and https://www.who.int/publications-detail/advice-on-the-use-of-masks-in-the-community-during-home-care-and-in-healthcare-settings-in-the-context-of-the-novel-coronavirus-(2019-ncov)-outbreak

Remote Postnatal Contacts Checklist

The following guidance is for remote postnatal contacts – standard practice should continue for all face-to-face visits. Where necessary, refer to local guidance on what should occur at routine postnatal contacts. This is not a comprehensive guide to content of postnatal visits – it is a guide to how remote visits might be structured.

Discuss support resources/network at home and any changes that may have occurred (i.e: family who can no longer travel to support the woman at home, childcare providers who are no longer available). Connect the woman to community support resources where available.

Discuss when and how the woman can contact their postpartum midwife or maternity care provider, especially in the case of an emergency.

Discuss family planning/birth spacing - all methods of contraception, including long acting reversible contraceptives, should be discussed in context of how provision of contraception may change within the limitations of decreased postpartum in-person visits. Discuss risks of failure of traditional methods of birth spacing. Discuss how and where to obtain contraceptive services if these have changed with COVID and aim to provide contraceptive of choice prior to discharge from health facility where feasible, or otherwise during postnatal face to face visits.

Remote Postnatal Contacts

Remote Postnatal Contacts Checklist
ALL contacts regardless of method
Respectful Maternity Care – includes:<br>✓ Treating all women with dignity and respect<br>✓ Maintaining confidentiality and privacy<br>✓ Freedom from discrimination<br>✓ Supporting women's right to information and informed autonomous decision making
Suggested actions<br>✓ Introduce yourself and greet the woman in a friendly manner<br>✓ Assessment for possible COVID-19 symptoms (both woman, baby and household contacts) and refer to country/facility guidance or pathway for care if symptoms identified<br>✓ Enquire about the woman's general health and wellbeing<br>✓ Consider physical, social, emotional and cultural wellbeing<br>✓ Enquire about the baby's wellbeing – include input, output and activity<br>✓ Undertake routine observation and assessment – both woman and baby<br>✓ Explain all assessments, tests and procedures and obtain consent<br>✓ Review pregnancy, birth and history to date and undertake ongoing assessment of risk factors<br>✓ Discuss newborn danger signs:<br>• stopped feeding well<br>• convulsions<br>• fast breathing (rate≥60/min)<br>• severe chest indrawing<br>• no spontaneous movement<br>• fever (temp ≥37.5)<br>• low body temperature (temp <35.5)<br>• any jaundice in first 24hrs or yellow palms/soles at any age<br>✓ Discuss maternal postnatal complications signs and symptoms:<br>• postpartum haemorrhage<br>• pre-eclampsia/eclampsia<br>• infection<br>• thromboembolism<br>• mastitis<br>✓ Discuss family planning/birth spacing<br>✓ Offer time for questions – take time to answer<br>✓ Provide individualised information and education<br>✓ Undertake consultation and referral where necessary<br>✓ Discuss plan for emergency transport from the woman's home to a health facility if needed<br>✓ Plan for next postnatal contact and ongoing care<br>✓ Document assessments, discussions and plans for continued care

Initial Face-to-Face Contact Birth – 24 hours	
✓ Routine initial assessments, education and discharge planning<br>✓ In addition to the standard first postnatal contact assessments/activities, need to ensure: Information about telehealth and schedule of postnatal contacts and obtain consent for phone/video calls.<br>✓ Confirm correct phone number for the woman and a backup phone number<br>✓ Ensure the woman has a contact number for midwife/practitioner providing remote postnatal contact or hospital/health service contact<br>✓ Ensure woman has enough iron, folic acid and other medications to help avoid facility-based postnatal contact just to obtain supplies	
Remote Contact Guidance - Modality can be swapped depending on individual woman's need	
Introduce self and friendly greeting<br>Respectful Maternity Care	☐
How are you feeling today?<br>Assessment for possible COVID-19 symptoms (both woman, baby and household contacts) and refer to country/facility guidance or pathway for care if symptoms identified	☐
Confirm date, time and mode of birth<br>Check if this is a first or subsequent child	☐
Were any problems identified during your pregnancy or at the time of the birth?<br>Ongoing risk assessment	☐
Emotional Wellbeing Assessment	
How are you recovering from the labour and birth?<br>Discuss labour and birth and allow debrief	☐
Are you getting some sleep?<br>Emotional assessment<br>Discuss newborn sleeping patterns	☐
Are you worrying a lot about anything?<br>Emotional assessment<br>Discuss coping strategies	☐
Tell me about your moods? Have you noticed any changes?<br>Emotional assessment<br>Discuss mood fluctuations and common postnatal feelings<br>Include early emotions – should be resolving by Day 10<br>Use depression screening tool if in use in context<br>Discuss signs/symptoms of Postnatal Depression and referral where appropriate	☐
Do you feel safe at home?<br>IPV/GBV assessment<br>Provide information on services and contact number (where available)	☐
Do you have some people who can provide you with support or help you if you need help?<br>Emotional /physical support<br>Safety planning	☐
Physical Wellbeing Assessment	
Are you eating and drinking well?<br>Consider dietary advice	☐
Are you taking any medications (Iron/parasite etc)?<br>Discuss routine supplementation/medications	☐
Are you worried about your bleeding? If Yes,<br>Tell me about how much bleeding you have?	☐

How often do you need to change your sanitary napkin?<br>What colour is the bleeding?<br>Are any clots present?<br>Does it have an unpleasant smell?<br>Discuss normal physiological changes postpartum and expected lochia loss up to 4-6 weeks after birth<br>Discuss when to seek urgent care for abnormal postpartum bleeding<br>If concerns, **consult or refer** as per usual practice/policy	
Are you having any trouble going to the toilet?<br>Urinary incontinence or retention<br>Constipation/Faecal incontinence	☐
Did you have any tears or stitches?<br>How does that area feel?<br>Rate pain scale 0-10 – (zero no pain, 10 extreme pain)<br>Are you able to walk and sit without too much pain?<br>What are you doing to keep the area clean?<br>Discuss perineal hygiene and care and pelvic floor exercises<br>If concerns, **consult or refer** as per usual practice/policy	☐
Are you mobilizing regularly?<br>Discuss venous thromboembolism prevention	☐
Have you noticed any danger signs/symptoms such as:<br>Increased vaginal bleeding or clots?<br>Smelly discharge?<br>Severe headache and/or blurred vision?<br>Fever and too weak to get out of bed?<br>Severe pain?<br>Fast or difficult breathing?<br>Flu-like symptoms with painful or reddened breasts?<br>Painful and/or swollen calves?<br>**If signs/symptoms present – consult or refer** as per usual practice/policy	☐
What would you do if you did have any of these signs/symptoms?<br>Include where and how to seek help	☐
Would you be able to get to the nearest health service? How?<br>Access to transport<br>Finances	☐
**Infant Feeding Assessment**	
Are you breastfeeding? If yes,<br>How are your breasts and nipples feeling?<br>Breasts – comfort, fullness, pain, redness.<br>Nipples – intact, cracked, bleeding. Discuss care of nipples and management of breast engorgement/hand expressing<br>Is the baby waking regularly for feeding?<br>Does the baby latch well and suck well? Discuss signs of a good latch and sucking pattern<br>How often does the baby breastfeed?<br>Discuss normal expected breastfeeding patterns for age (48-72 hours, 7-14 days and around 6 weeks)<br>How many wet/dirty nappies (diapers) has the baby had in the last 24 hours?<br>Discuss normal output for age<br>Are you keeping your baby skin-to-skin as much as possible?<br>Discuss benefits of skin-to-skin	☐

Discuss importance of exclusive breastfeeding and strategies to ensure ongoing adequate milk supply<br>Provide information and contact details on community services for support with breastfeeding (if available)	
If formula feeding:<br>Discuss importance of handwashing before formula preparation, sterilising equipment and following instructions for mixing formula with clean drinking water<br>If relevant, discuss initiation of breastfeeding if woman is open to discussion or if delaying initial breastfeeding due to culturally influenced beliefs	☐
**Newborn Assessment**	
How is your baby going?<br>General newborn wellbeing.<br>Discuss normal infant behaviours/sleep patterns and settling techniques	☐
Are you worried about anything to do with your baby?<br>Maternal assessment of baby	☐
Have you noticed any of the following danger signs?<br>Has your baby stopped feeding well?<br>Has your baby's movements or activity levels decreased?<br>Has your baby had any convulsions/seizures?<br>Is your baby difficult to wake up or very sleepy all the time?<br>Has your baby felt too hot or too cold for no reason?<br>Discuss appropriate clothing – 1 or 2 more layers than adults only<br>Have you noticed your baby breathing much faster than usual or does baby's chest draw-in when breathing?<br>Does your baby have yellow discolouration of baby's palms or soles of feet?<br>If Danger Signs present – consult or refer as per usual practice/policy<br>Discuss seeking health care early if they notice any of these signs	☐
Newborn feeding and output - addressed above in Infant Feeding	☐
What does your baby's cord stump look like?<br>Discuss cord care if advised in your setting	☐
Discuss importance of hand washing and general hygiene in preventing infections in newborns	☐
Consider any maternal conditions that may require additional care/treatment in the newborn, such as:<br>HIV<br>Untreated Syphilis or other STIs in pregnancy	☐
Give opportunity to discuss any concerns/questions	☐
**Health Promotion/Education Topics**	
Consider Health Promotion/Education counselling<br>Exclusive Breastfeeding<br>Discuss common issues such as concerns regarding not enough milk, ensuring adequate supply and breast health<br>Integrate Lactational Amenorrhoea Method (LAM) criteria<br>Family planning and Birth Spacing<br>LAM as above<br>Discuss modern methods and contraception options<br>Ensure desired method commenced or provided before discharge after birth or during postpartum health facility contact. Ensure appropriate referral if contraceptive methods not available at time of postpartum contact.<br>Discuss resumption of sexual relations and safe sex<br>Nutrition information	☐

Context specific counselling such as:<br>Malaria precautions	
Depending on schedule of contacts:<br>Make next appointment	☐
Do you have any other questions? Or Is there anything you want to talk about?	☐
Remind the woman of importance of postnatal care, keeping her next postnatal contact and the process to follow if she has concerns regarding herself or her baby.	☐
Consider:<br>Have you identified any new risk factors? If so,<br>Does this woman need a face-to-face postnatal contact?<br>Does this woman know the referral pathway for accessing hospital services during COVID-19?<br>How/where will you document this contact?	

Reference List

1. RCOG & RCM, *Coronavirus (COVID-19) Infection in Pregnancy. Information for health care professionals. Version 5, published Saturday 28th March 2020*. 2020, Royal College of Obstetricians and Gynaecologists and Royal College of Midwives: London.
2. RCOG & RCM, *Guidance for antenatal and postnatal services in the evolving coronovirus (COVID-19) pandemic: Information for health professionals*. 2020, Royal College of Obstetricians & Gynaecologists and The Royal College of Midwives.
3. UNFPA, *Coronavirus Disease (COVID-19) Preparedness and Response UNFPA Interim Technical Brief* 2020, UNFPA: New York.
4. International Confederation of Midwives (ICM), *Women's Rights in Childbirth Must be Upheld During the Coronavirus Pandemic*. 2020, International Confederation of Midwives: The Hague.
5. World Health Organization. *Q&A on COVID-19, pregnancy, childbirth and breastfeeding*. 2020 [cited 2020 1st April]; Available from: https://www.who.int/news-room/q-a-detail/q-a-on-covid-19-pregnancy-childbirth-and-breastfeeding.
6. Queensland Health, *Queensland Clinical Guidelines Perinatal care of suspected or confirmed COVID-19 pregnant women. Guideline No. MN20.63-V1-R25.* . 2020, State of Queensland (Queensland Health) Queensland, Australia.
7. The White Ribbon Alliance. *Respectful Maternity Care Charter*. 2011 [cited 2020 03/04/2020]; Available from: https://www.whiteribbonalliance.org/respectful-maternity-care-charter/.
8. Sandall, J., et al., *Midwife-led continuity models versus other models of care for childbearing women.* Cochrane Database of Systematic Reviews, 2016(4).
9. World Health Organization, *WHO Recommendations on the Postnatal Care of the Mother and Newborn*. 2013, World Health Organization: Geneva.
10. Healthy Newborn Network (HNN). *Healthy Newborn Network: PNC Checklist*. 2020 [cited 2020 21 April]; Available from: https://www.healthynewbornnetwork.org/hnn-content/uploads/PNC-Checklist_Asia-1.pdf
11. World Health Organization, *Advice on the use of masks in the context of COVID-19*. 2020, World Health Organization: Geneva.
12. World Health Organization, *Rational use of personal protective equipment (PPE) for coronavirus disease (COVID-19) Interim Guidance*. 2020, World Health Organization: Geneva.
