# Supplementary material for: Midwife-led pandemic telemedicine services for maternal health and gender-based violence screening in Bangladesh: an implementation research case study
Source: Reprod Health. 2023 Aug 29;20:128. doi: 10.1186/s12978-023-01674-0 (PMC10466754; doi:10.1186/s12978-023-01674-0)

CALENDAR

FOR REMOTE CONTACT MATERNITY SERVICES DURING COVID 19

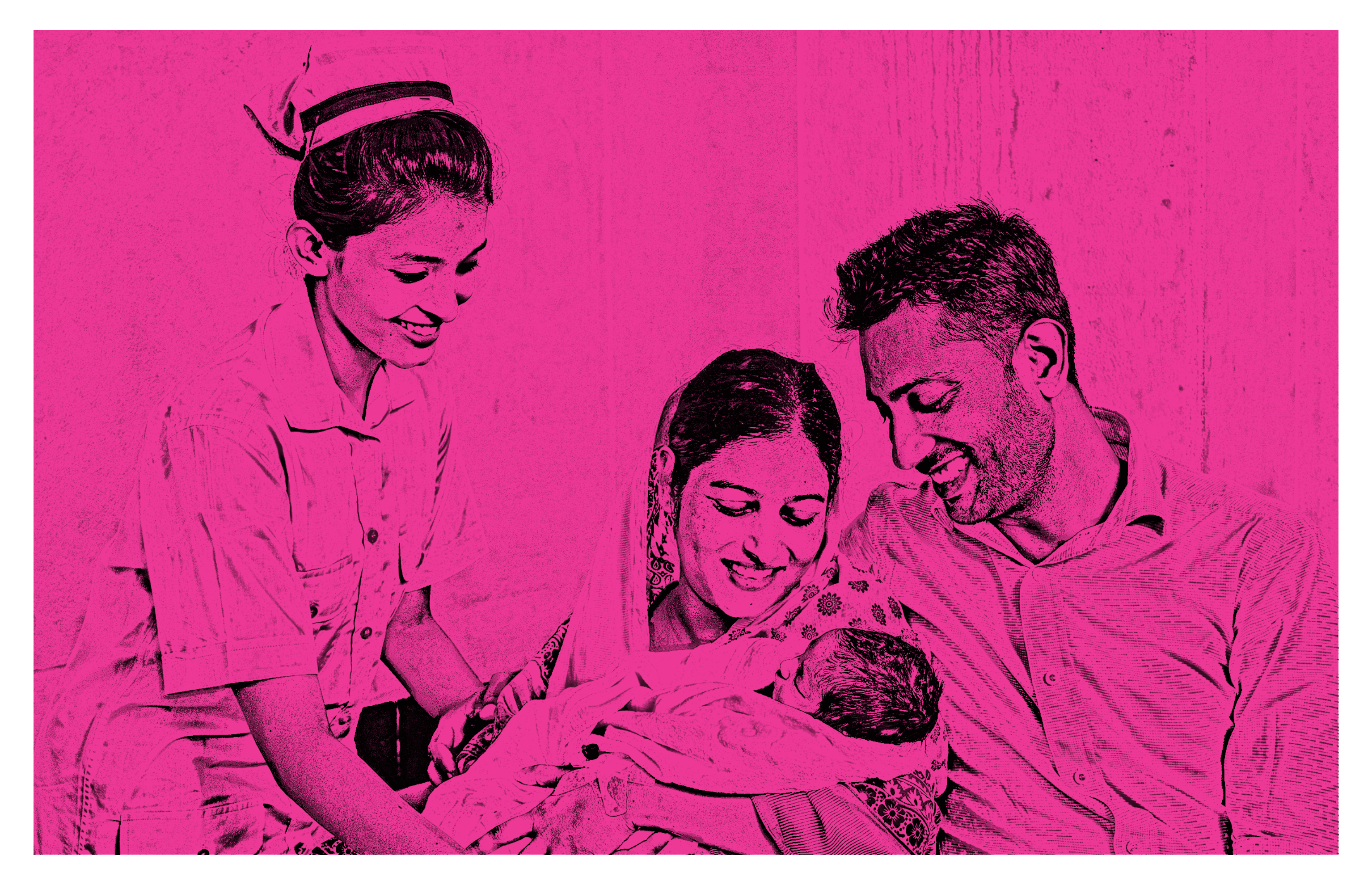

DIRECTORATE GENERAL OF NURSING AND MIDWIFERY

January-2021

SUN	MON	TUE	WED	THU	FRI	SAT
31					1	2
3	4	5	6	7	8	9
10	11	12	13	14	15	16
17	18	19	20	21	22	23
24	25	26	27	28	29	30

"Babies are bits of star-dust blown from the hand of God.
Lucky is the woman who knows the pangs of birth for she has held a star."
– Larry Barretto

February-2021

SUN	MON	TUE	WED	THU	FRI	SAT
	1	2	3	4	5	6
7	8	9	10	11	12	13
14	15	16	17	18	19	20
21	22	23	24	25	26	27
28						

"Babies are bits of star-dust blown from the hand of God.
Lucky is the woman who knows the pangs of birth for she has held a star."
– Larry Barretto

March-2021

SUN	MON	TUE	WED	THU	FRI	SAT
	1	2	3	4	5	6
7	8	9	10	11	12	13
14	15	16	17	18	19	20
21	22	23	24	25	26	27
28	29	30	31			

"Babies are bits of star-dust blown from the hand of God.
Lucky is the woman who knows the pangs of birth for she has held a star."
– Larry Barretto

April-2021

SUN	MON	TUE	WED	THU	FRI	SAT
				1	2	3
4	5	6	7	8	9	10
11	12	13	14	15	16	17
18	19	20	21	22	23	24
25	26	27	28	29	30	

“Babies are bits of star-dust blown from the hand of God.
Lucky is the woman who knows the pangs of birth for she has held a star.”
– Larry Barretto

May-2021

SUN	MON	TUE	WED	THU	FRI	SAT
30	31					1
2	3	4	5	6	7	8
9	10	11	12	13	14	15
16	17	18	19	20	21	22
23	24	25	26	27	28	29

"Babies are bits of star-dust blown from the hand of God.
Lucky is the woman who knows the pangs of birth for she has held a star."
– Larry Barretto

June-2021

SUN	MON	TUE	WED	THU	FRI	SAT
		1	2	3	4	5
6	7	8	9	10	11	12
13	14	15	16	17	18	19
20	21	22	23	24	25	26
27	28	29	30			

"Babies are bits of star-dust blown from the hand of God.
Lucky is the woman who knows the pangs of birth for she has held a star."
– Larry Barretto

July-2021

SUN	MON	TUE	WED	THU	FRI	SAT
				1	2	3
4	5	6	7	8	9	10
11	12	13	14	15	16	17
18	19	20	21	22	23	24
25	26	27	28	29	30	31

"Babies are bits of star-dust blown from the hand of God.
Lucky is the woman who knows the pangs of birth for she has held a star."
– Larry Barretto

August-2021

SUN	MON	TUE	WED	THU	FRI	SAT
1	2	3	4	5	6	7
8	9	10	11	12	13	14
15	16	17	18	19	20	21
22	23	24	25	26	27	28
29	30	31				

“Babies are bits of star-dust blown from the hand of God.
Lucky is the woman who knows the pangs of birth for she has held a star.”
– Larry Barretto

September-2021

SUN	MON	TUE	WED	THU	FRI	SAT
			1	2	3	4
5	6	7	8	9	10	11
12	13	14	15	16	17	18
19	20	21	22	23	24	25
26	27	28	29	30		

“Babies are bits of star-dust blown from the hand of God.
Lucky is the woman who knows the pangs of birth for she has held a star.”
– Larry Barretto

October-2021

SUN	MON	TUE	WED	THU	FRI	SAT
31					1	2
3	4	5	6	7	8	9
10	11	12	13	14	15	16
17	18	19	20	21	22	23
24	25	26	27	28	29	30

*"Babies are bits of star-dust blown from the hand of God.
Lucky is the woman who knows the pangs of birth for she has held a star."*
– Larry Barretto

November-2021

SUN	MON	TUE	WED	THU	FRI	SAT
	1	2	3	4	5	6
7	8	9	10	11	12	13
14	15	16	17	18	19	20
21	22	23	24	25	26	27
28	29	30				

"Babies are bits of star-dust blown from the hand of God.
Lucky is the woman who knows the pangs of birth for she has held a star."
– Larry Barretto

December-2021

SUN	MON	TUE	WED	THU	FRI	SAT
			1	2	3	4
5	6	7	8	9	10	11
12	13	14	15	16	17	18
18	19	20	22	23	24	25
26	27	28	29	30	31	

"Babies are bits of star-dust blown from the hand of God.
Lucky is the woman who knows the pangs of birth for she has held a star."
– Larry Barretto

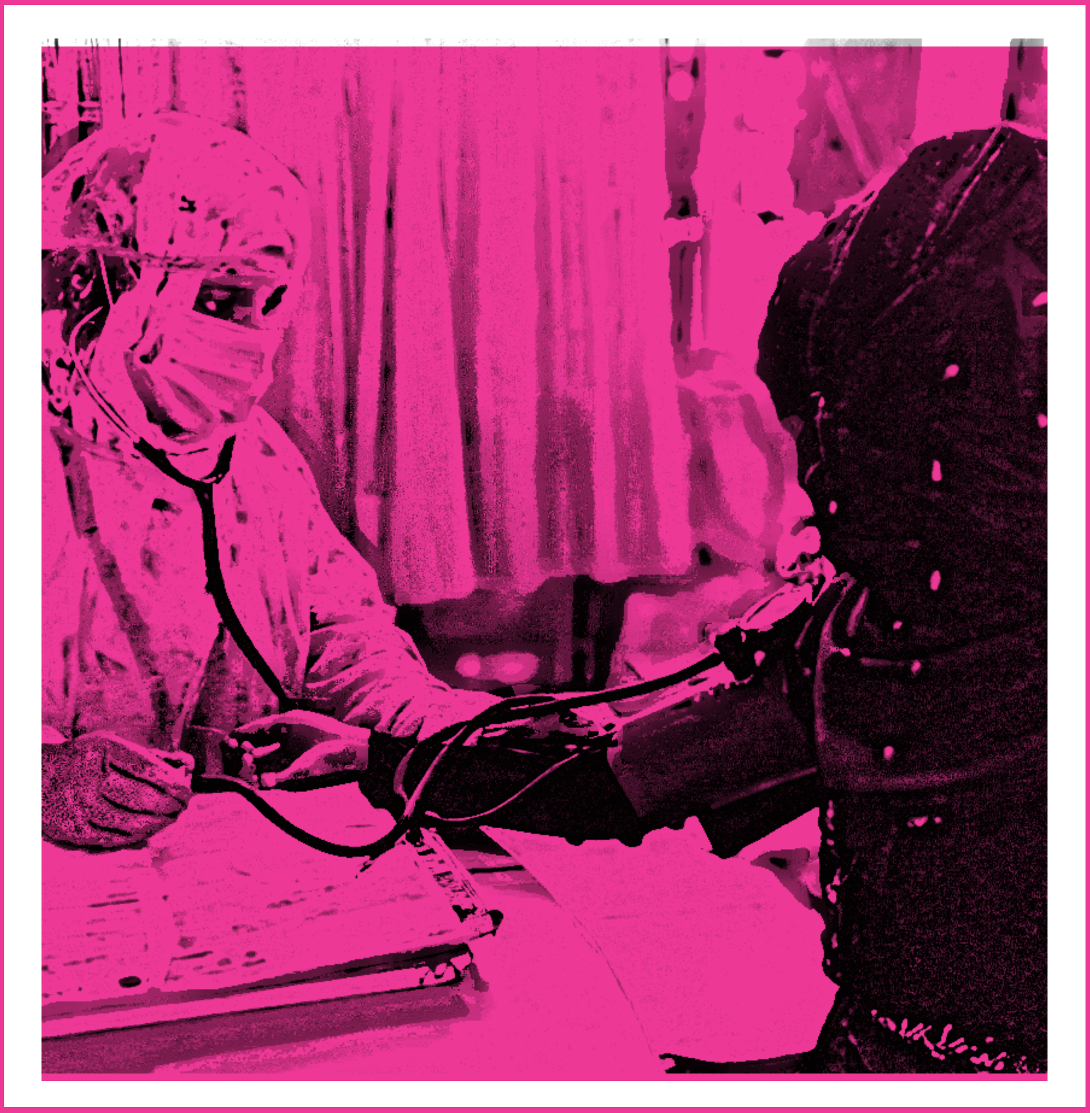

Supplement: Supplementary file 3 — Additional file 3: Version 1 of the scheduling tool used in the telemedicine intervention. [file 12978_2023_1674_MOESM3_ESM.pdf]
