# Supplementary material for: Midwife-led pandemic telemedicine services for maternal health and gender-based violence screening in Bangladesh: an implementation research case study
Source: Reprod Health. 2023 Aug 29;20:128. doi: 10.1186/s12978-023-01674-0 (PMC10466754; doi:10.1186/s12978-023-01674-0)
Supplement: Supplementary file 4 [file 12978_2023_1674_MOESM4_ESM.pdf]

2 July | Saterday | 2022
